# Interventions for promoting physical activity in people with newly diagnosed Parkinson’s disease: scoping review

**DOI:** 10.1186/s13643-025-02892-2

**Published:** 2025-08-09

**Authors:** Jonathan Gilby, Bridie Kent, Rachel Knight Lozano, Jonathan Marsden

**Affiliations:** 1 School of Health Professions, Faculty of Health, University of Plymouth, Plymouth, UK; 2 School of Nursing and Midwifery, Faculty of Health, University of Plymouth, Plymouth, UK

**Keywords:** Interventions, Newly diagnosed Parkinson’s, Physical activity, Scoping review

## Abstract

**Background:**

There is increasing evidence to suggest that physical activity can slow Parkinson’s progression. There is also increasing interest in non-pharmacological interventions to alleviate Parkinson’s symptoms. This scoping review aimed to map and describe the evidence for interventions that promote physical activity in people with newly diagnosed Parkinson’s.

**Methods:**

Studies conducted since 2011, on adults with Parkinson’s (≥ 18 years), investigating the effects of non-pharmacological interventions to promote physical activity and/or exercise were considered. Interventions needed to be conducted in healthcare or healthcare-related settings for people within 5 years of Parkinson’s diagnosis. Published or unpublished full-text articles since 2011 were searched in November 2023, using online focused, broad, and grey literature databases. JBI scoping review methodology was used and results presented in table format accompanied by a narrative review.

**Results:**

A total of 22 articles with a variety of research designs were included with 14 randomized trials, one single-site, prospective, single-arm study, two retrospective cohort studies, one case series, two case reports, and two qualitative reports. Many studies (*n* = 7) were conducted in outpatient clinics with the majority of interventions (*n* = 17) involving physiotherapists. Interventions varied widely, including aerobic exercise, balance exercise, dance, and yoga. The duration of intervention varied from 4 weeks to 8 years. Dosage of interventions varied widely from 30 to 90 min, and from twice weekly to seven times weekly. Several different outcome measures related to physical activity levels and/or physical fitness were used. The most frequent clinician/researcher reported outcome measure was the 6-min walk test (in nine studies) and the most frequently used participant/patient reported outcome measure was the 39-item Parkinson’s Disease Questionnaire (PDQ-39) (also in nine studies).

The review showed limited research in identifiable cohorts with newly diagnosed Parkinson’s. Sample sizes were predominantly small. In all but one study, authors interpreted their results as favoring interventions to promote physical activity for people with newly diagnosed Parkinson’s. All authors recommended further studies.

**Conclusions:**

There is a need for more research with larger sample sizes and standardized reporting to inform the evidence base for interventions that promote physical activity in people with newly diagnosed Parkinson’s.

**Systematic review registration:**

https://pearl.plymouth.ac.uk/ (http://hdl.handle.net/10026.1/20098)

**Supplementary information:**

The online version contains supplementary material available at 10.1186/s13643-025-02892-2.

## Background

Parkinson’s disease (Parkinson’s) is a progressive neurological condition that causes motor and non-motor symptoms. The cause is believed to involve both genetic and environmental factors [1]. Parkinson’s occurs more commonly in men than in women and in those over the age of 60 years [2]. Prevalence is rising more rapidly than in other neurological disorders and, for example, in the UK prevalence is predicted to rise by 23.2% to 168,582 people with Parkinson’s (pwp) by the year 2025 [2, 3].

Common impairments in Parkinson’s are slow movement, balance problems, rigidity, tremor, apathy, low mood, sleep behaviour disorder, and memory problems. These can lead to participation and activity limitations and affect quality of life [3–5]. It is thought that these combined impairments contribute to pwp leading more sedentary lives than age-matched controls [6, 7].

Physical activity is any bodily movement produced by skeletal muscles that results in a substantial increase over resting energy expenditure, with exercise being a subset of physical activity that is planned, structured and repetitive [8]. Increased physical activity in older adults is associated with lower rates of chronic disease, healthier body composition and bone health, lower falls risk, greater levels of independence, and better cognitive functioning [9]. Additional to these generic benefits, there is mounting evidence that aerobic exercise improves physical fitness and attenuates motor symptoms for pwp [10]. Further, there are indications from animal and human models that it may have a preventative and disease-modifying effect through mechanisms such as increased dopamine metabolism, neurogenesis, angiogenesis, and increased brain-derived neurotrophic factor [11–15].

The mainstay of Parkinson’s treatment for the last several decades has been levodopa medication but this has limitations and cannot address the full range of motor and non-motor symptoms that impact quality of life for people with Parkinson’s [16, 17]. Early delivery of physical training is emphasized by the recent Parkinson’s Foundation task force highlighting that physical activity not only improves physical outcomes but may also benefit mental health problems [18]. It may also improve other Parkinson’s symptoms such as insomnia or constipation [19]. However, pwp have been shown to do less physical activity than age-matched controls and when measured against World Health Organisation (WHO) physical activity guidelines [6, 7]. Increased awareness of neurophysiological effects and levels of physical activity and exercise in pwp has led to a number of guidelines published since 2011, which have recommended interventions that promote physical activity in pwp with a resultant increase in research activity in this area [3, 20, 21].

Therapy, particularly physiotherapy, has been proposed to promote healthy physical activity and exercise to prevent functional decline [22]. Clinical practice guidelines for pwp in Europe, USA, and the UK recommend specialized therapy soon after diagnosis for physical activity support, individualized interventions, and education [3, 20, 21]. However, therapy for newly diagnosed pwp remains under-utilized. The 2022 Parkinson’s UK audit identified that only 25.3% of Parkinson’s patients surveyed (*n* = 1837) were referred to physiotherapy and only 32.1% (*n* = 627) were referred to occupational therapy within the first 3 years post-diagnosis [23].

A recent scoping review of physical self-management for pwp identified a gap between what the evidence promotes and what is being achieved. Further, the authors suggested that research should focus on and elucidate the type, intensity, amount, and duration of physical self-management models [24]. Most trials have been conducted in pwp who have already developed motor symptoms but the evidence for non-pharmacological early interventions to prevent the onset or reduce the severity of symptoms is an important research area that needs to be explored [20].

Defining early intervention in Parkinson’s is challenging due to difficulties in defining newly diagnosed Parkinson’s. Hoehn and Yahr (H&Y) staging is often used in research and thought to correlate with time from disease onset [25]. Its primary use is, however, as a measure of disease severity and a criticism is non-linearity whereby pwp may not go through stages sequentially [25, 26]. ‘De novo’ has been used in studies as a term to define people with newly diagnosed Parkinson’s that are drug naïve for levodopa or dopamine agonists [27, 28]. Changes to prescribing practices whereby people are prescribed levodopa much sooner means that the term ‘newly diagnosed’ can encompass both patients not receiving levodopa or early Parkinson’s [29]. Further, there is long-standing debate over length of prodromal phase, disease onset, and time to diagnosis [30, 31]. Healthcare input within time from diagnosis is used as a benchmark monitoring quality standards in Parkinson’s care [23] and can be seen in the baseline characteristics described in many research studies.

Movement Disorder Society Criteria for Clinically Established Early Parkinson’s has previously used 5 years disease duration to aid in the development of its criteria [32–34]. Using the alternative cutoff of 5 years from diagnosis takes into account baseline characteristic reporting in many research studies and the potential for delays in referral-to-treatment in healthcare systems, for example, a large study identified that the time to final clinical diagnosis of Parkinson’s in 95% of patients was 2.75 years [31]. Using 5 years from diagnosis also mitigates against the potential confounding variable of mis-diagnosis within the first 5 years of symptom duration [34].

There are various interventions and service delivery approaches that may or may not be applicable to the needs of people with newly diagnosed Parkinson’s. The scoping review approach lends itself well to exploring the interventions that have been applied across the world specifically to those with newly diagnosed Parkinson’s. With the aim of identifying the types of evidence available and providing an overview of what is currently known in the research area, this review meets a key indication for scoping reviews [35].

The over-arching aim of this review was to assess the extent of the literature in the field of interventions to promote physical activity in those with newly diagnosed Parkinson’s in healthcare and healthcare-related settings.

### Review question(s)

 The Population, Concept, and Context (PCC framework) for the current scoping review are people with newly diagnosed Parkinson’s (and related terms for this group) and interventions for promoting physical activity (and terms related to physical activity) in healthcare (and related) settings (e.g., free-living or laboratory environments where the research conducted is linked to participant healthcare provision).

 The review question is: What interventions are used in the promotion of physical activity in people with newly diagnosed Parkinson’s in healthcare and healthcare-related settings?

Sub-questions:

1. What are the baseline characteristics of the populations being measured?
2. What are the characteristics of the professionals providing the interventions?
3. What settings are used to provide the interventions?
4. What methodology and outcome measures are used to determine effects of interventions?
5. What are the key findings and recommendations from studies looking at these interventions?

## Methods

This scoping review was conducted in accordance with JBI methodology for scoping reviews [36] and reported in line with the Preferred Reporting Items for Systematic Reviews and Meta-Analyses Extension for Scoping Reviews (PRISMA-ScR) Checklist (see Appendix A). The objectives, inclusion criteria, and methods for this scoping review were specified in advance and are publicly available in the University of Plymouth research repository (https://pearl.plymouth.ac.uk/).

### Inclusion criteria

#### Participants

 This review considered studies that included adults (over 18 years of age) with newly diagnosed Parkinson’s (within 5 years of diagnosis). Parkinson-Plus syndromes and Juvenile Parkinson’s Disease are excluded.

#### Concept

 This review considered studies exploring non-pharmacological interventions to promote physical activity and/or exercise. It reports on measures related to changes in physical activity/planned physical activity/exercise/fitness, including measures of impairment, activity or participation relating to physical activity/fitness levels. Both clinician-reported or patient/caregiver-reported outcome measures in free-living and/or laboratory environments are included.

#### Context

 This review considered studies conducted in healthcare and healthcare-related research settings.

#### Other criteria

 Full text articles available since 2011 in English language. Quantitative, qualitative, or mixed-methods studies with no limit on type of study or publication status as the purpose of this review was to map the evidence rather than produce a critically appraised and synthesized result with an assessment of methodological limitations or risk of bias. This was in line with scoping review guidance on reviews of this type [35, 36].

### Types of sources

 This scoping review considered a broad range of research methods together with text and opinion including randomized controlled trials, non-randomized controlled trials, before and after studies, and interrupted time-series studies. Additionally, analytical observational studies including prospective and retrospective cohort, case-control, and analytical cross-sectional studies were considered for inclusion. This review also considered for inclusion descriptive observational study designs, including individual case reports, case series, and descriptive cross-sectional studies.

 Qualitative studies were also considered that focus on qualitative data including, but not limited to, designs such as qualitative description, grounded theory, ethnography, phenomenology, and action research. Systematic reviews that met the inclusion criteria were also considered, depending on the research question and if they provided sufficient detail on the interventions provided in the primary research. Text and opinion papers were considered for inclusion if they provided details on the physical activity interventions provided.

### Search strategy

 A three-step search strategy was utilized, following an initial search of MEDLINE, the Cochrane Database of Systematic Reviews and *JBI Evidence Synthesis *to confirm that no similar review had been completed or was underway. The first-step was an initial limited search of online databases relevant to the research area (Ovid MEDLINE, EBSCOhost CINAHL). This identified appropriate relevant articles and key words to develop the full search strategy for the second stage of the search strategy. The search strategy was updated for each included database following input from a University Information Specialist. Search strings are available from https://searchrxiv.org/. Published or unpublished full-text articles written in English since 2011 were searched using online broad databases and grey literature databases on September 17, 2021. Databases included MEDLINE, EMBASE, CINAHL, AMED; the online broad databases of PubMed, Scopus; and grey literature databases of OpenGrey, EthOS, Google Scholar. To update the results to incorporate more recent articles, a search of the MEDLINE, CINAHL, EMBASE, AMED, SCOPUS, and Google Scholar databases was repeated between November 14, and November 22, 2023. Thirdly, the reference lists of identified reports and articles identified for full-text review were searched by two independent reviewers (JG and JM).

Only studies with an identifiable cohort of participants within 5 years of Parkinson’s diagnosis were considered. The cohort mean or median years since diagnosis was accepted as evidence of this (standard deviations and confidence intervals given where provided). Only studies published since 2011 were considered due to developments in the field in this timeframe following the recommendations of a number of reviews and guidelines [3, 14, 15, 20, 37–39]. Full text articles only were included. Further, only sources of evidence published in English were considered for inclusion due to the first language of the review team.

### Study/source of evidence selection

All retrieved articles were uploaded to Mendeley reference management software (Mendeley 1.19.8 Mendeley Ltd., Elsevier, Netherlands); duplicates were identified and removed before transfer to Rayyan 2022 software (Rayyan Systems Inc., MA, USA).

All titles and abstracts were uploaded to Rayyan for independent screening. Applying the inclusion criteria, initial title screening was conducted by the primary author (JG). Abstract screening was then conducted by independent researchers (JG, RKL, and JM). Attempts were made to obtain full texts of selected articles by web searching and engaging with University of Plymouth Information Specialists.

Three independent researchers (JG, RKL, and JM) conducted full-text screening of selected studies. A fourth reviewer (BK) was on-hand for discrepancies that could not be resolved by discussion and consensus, but this proved unnecessary. Exclusions were documented according to the eligibility flowchart (see Appendix B). Reference screening of full text articles was conducted by JG and JM using the same screening process.

### Data extraction

 Data were extracted from included papers by three independent reviewers (JG, RKL, and JM) using a data extraction tool in line with JBI scoping review methodology [40]. A fourth reviewer (BK) was on hand to support, but any disagreements that arose between reviewers were resolved through discussion. An extraction table was developed by the reviewers and adapted from the JBI manual [36]. As the data extraction tool worked well, further refinement proved unnecessary after initial piloting. The extraction table and results for the included articles are available on request from the lead author.

### Data analysis and presentation

Results are reported graphically with tables where possible with an accompanying narrative to further describe the literature. The findings of the review are reported in relation to the review question and sub-questions, reflecting the objectives of the review (see above).

## Results

**Fig. 1:**
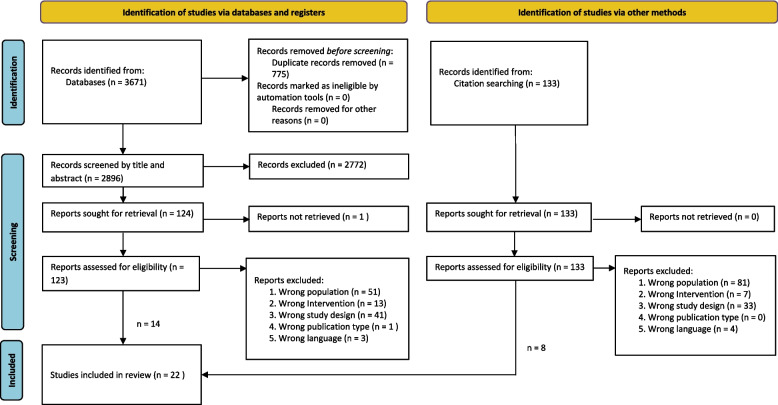
Search results and source selection and inclusion process. PRISMA flow diagram

The results of the screening process can be seen below in Fig. 1. 

### Search results

 A total of 3671 titles were identified and uploaded to Mendeley. Of these, 775 were duplicates. At the title and abstract phases, 2896 articles were screened with 2772 articles ineligible. There were 124 full-text articles assessed for eligibility and 110 excluded (see Fig. 1).

 Reasons for exclusion at the full-text stage were as follows: wrong population criteria (*n* = 51), wrong intervention criteria (*n* = 13), wrong study design criteria (*n* = 41), wrong publication type (*n* = 1), and wrong language (*n* = 3). One study was unobtainable because the poster presentation had been withdrawn from the journal by the author [41].

 An examination of the reference lists of the included papers provided a further 133 articles for review, with 125 excluded (see Fig. 1), resulting in 22 papers being reviewed.

### Review findings

The main review characteristics findings are presented in Tables 1 and 2. A further study characteristics table can be found in Supplementary Table 1, Additional File 1. The accompanying narrative review of the evidence has sub-headings relating to the review question and sub-questions described above.

**Table 1:** Description of included studies with interventions to promote physical activity in people with newly diagnosed Parkinson’s

Author, date and country	Intervention	Brief description, intervention intensity (where stated) and intervention duration
**Bang and Shin (2017). (1) Republic of Korea.**	Intensive Nordic walking intervention effect on the balance function and walking ability of individuals with PD.	Single-blinded RCT, with the assessor blinded to treatment. 20 participants allocated to Nordic walking on treadmill (NWT) or treadmill walking only (TT). High intensity. The training program consisted of 20, 60 min sessions, performed 5 days per week, for 4 weeks. Physiotherapist delivered.
**Cancela-Carral et al. (2022). (2) Spain**	Monitoring the level of physical fitness and anthropometric parameters of patients diagnosed with Parkinson’s Disease, who had participated in physical activity programs arranged by the Parkinson’s Disease Association of Pontevedra	Eight year observational retrospective study. Intensity not recorded. Land exercise program: 2 × 60 min sessions per week including 10 min warm-up, aerobic exercises with strength, flexibility, joint mobility and co-ordination tasks, 5 min cool down. Aquatic exercise program: 1 × 60 min session per week including 10 min warm-up, 20 min static exercises, 20 min dynamic exercises, 10 min cool down. (From 2015 onwards) Pilates program 2 × 60 min sessions including 10 min warm-up, 45 min strength, stability and co-ordination exercises, 5 min cool down. Delivered by health professionals (physiotherapy graduates, physical activity, and sports science graduates)
**Carda et al. (2012). (3) Switzerland**	Evaluation of Lokomat robotic treadmill gait training compared to conventional treadmill gait training in patients with PD.	Randomized, single-blind controlled trial. 30 participants, 14 intervention group, 14 control group (1 loss to follow-up in each arm). Lokomat robotic gait training on treadmill for 30 min (50% body-weight support) for 15 min and 30% body weight support for 15 min 3 days a week for 4 weeks; Control Group: 30 minutes of gait training on a treadmill 3 days a week for 4 weeks. Moderate to high intensity: Patients were trained using 80% of the maximum speed they had reached during the test for the first week, 90% for the second week, and 100% for the third and fourth weeks. Patients were evaluated by a physical therapist blinded to allocation before and at the end of treatment and then at the 3- and 6-month follow-up.
**Clarke et al. (2016). (4) UK**	Clinical effectiveness and cost-effectiveness of physiotherapy and occupational therapy versus no therapy in mild to moderate PD.	A large pragmatic RCT (PD REHAB) performed in 38 neurology and geriatric medicine outpatient clinics in the UK. Patients were randomized online to either both physiotherapy and occupational therapy NHS services (*n* = 381) or no therapy (*n* = 381). Therapy incorporated a patient-centered approach with individual assessment and goal setting. Median number of therapy sessions: 4; Mean time per session 58 min; Mean duration of therapy 8 weeks. Intensity not recorded.
**Ellis et al. (2019). (5) USA**	Comparative effectiveness of mobile health (mHealth)-supported exercise compared with exercise alone for people with PD.	Randomized Controlled Pilot Study: The purpose of this study was to explore the preliminary effectiveness, safety, and acceptability of a mHealth-mediated exercise program designed to promote sustained PA in people with PD (Walking with a pedometer plus engagement in planned exercise supported by mHealth (*n* = 23)) compared over 1 year with an active control condition (Walking with a pedometer and exercise only (*n* = 21)). Moderate intensity. Participants were asked to do 5 to 7 exercises for ≥ 3 days a week for 1 year.
**Fishel et al. (2020). (6) USA**	The impact of LSVT BIG therapy on postural control for individuals with PD	The purpose of this case series was to describe the impact of LSVT BIG therapy on postural control and gait in 3 individuals with moderate PD (1 met inclusion criteria for scoping review). The LSVT BIG individual training consists of Maximal Daily Exercises, Functional Component Tasks, BIG Walking, and Hierarchy Tasks. High intensity. Patients are prescribed LSVT BIG daily home exercises to complete once per day on treatment days and twice per day on non-treatment days; Each patient was scheduled for 16, 60 min intervention sessions provided 4 days per week for four consecutive weeks delivered by physiotherapists or occupational therapists.
**Frazzitta et al. (2015). (7) Italy**	Intensive Rehabilitation Treatment in Early PD.	2-year follow-up study to determine whether intensive exercise in the early stages of the disease slows down PD progression. Random assignment, parallel-group, pilot study with a 1:1 allocation ratio.High intensity.Intervention group (*n* = 20, 16 analyzed) 2, 28 day Multidisciplinary Intensive Rehabilitation Treatments (MIRT) (3 × 60 min daily sessions, 5 days a week), 1 year apart. Control group–medication only (*n* = 20, 15 analyzed).
**Handlery et al. (2021). (8) USA**	Effects of high-intensity treadmill exercise, moderate-intensity treadmill exercise, or usual care on people with de novo PD.	Secondary analysis of the Study in Parkinson’s Disease of Exercise trial: randomized participants to high-intensity treadmill exercise, moderate-intensity treadmill exercise, or usual care. Daily steps and MVPA assessed at baseline and once each month using an activity monitor. Fitness was assessed via graded exercise test at baseline and at 6 months. A step threshold that corresponds to meeting PA guidelines was determined by receiver operating characteristic curves.High-intensity treadmill exercise (4 days per week, 80–85% maximum heart rate (*n* = 43), moderate-intensity treadmill exercise (4 days per week, 60–65% maximum heart rate (*n* = 45), or wait-list control (*n* = 40) for 6 months.
**van der Kolk et al. (2019). (9) Netherlands.**	Effectiveness of home-based and remotely supervised aerobic exercise in PD.	A double-blind, RCT. Aimed to evaluate the effectiveness of aerobic exercise–gamified and delivered at home, to promote adherence - on relieving motor symptoms in patients with PD with mild disease severity who were on common treatment regimes. 130 randomly assigned to either the aerobic intervention group (n=65) or the active control group (*n* = 65). High intensity intervention group:30–45 min training, three times per week for 6 months.
**Landers et al. (2019). (10) USA**	A High-Intensity Exercise Boot Camp for Persons with PD.	A Phase II, Pragmatic, Randomized Clinical Trial of feasibility, safety, signal of Efficacy, and disease mechanisms with participants were randomized into one of two 8-week arms: High-Intensity multimodal exercise Boot Camp (HIBC) (aerobic, strengthening, and balance training, *n* = 14); or Usual Care (UC) (*n* = 13).
**Leavy et al. (2019). (11) Sweden**	Perceptions of balance and falls following a supervised training intervention.	A qualitative study of people with PD. Purposive sampling (13 people with Parkinson's - 8 identifiable as < 5 years since diagnosis), Individual semi-structured interviews by 1 author 70–95 min in duration. Interviews 3–12 months following participation in a 10 week supervised balance training program (Hi-Balance–performed in groups of 4 to 7 participants, 3 times per week, 60 min per session, for 10 weeks at a university hospital supervised by physiotherapists. Components of: sensory integration; anticipatory postural adjustments; motor agility; stability limits). Described as ‘highly challenging’ but intensity not reported.
**Li et al. (2020). (12) Australia**	Evaluation of the impact of an early intervention program on exercise behavior and mood in people with PD.	Retrospective cohort study with 1-year longitudinal follow-up including people with PD who enrolled in the 5 week PD Wellbeing Program at Calvary Hospital from August 2013 to September 2015 (*n* = 152). Intensity not reported.
**Moriello et al. (2013). (13) USA**	Incorporating yoga into an intense physical therapy program in someone with PD.	The purpose of this case report was to document outcomes following an intense exercise program integrating yoga with physical therapy exercise in a male with PD. High intensity. Intense 90 min program (Phase A) incorporating strengthening, balance, agility, and yoga exercises twice weekly for 12 weeks. Then completed a new home exercise program developed by the researchers (Phase B) for 12 weeks.
**Nero et al. (2016). (14) Sweden**	Effects of balance training on habitual PA and sedentary behavior in older adults with PD (Thesis Paper III).	Paper III described a RCT with short- and long-term follow up, evaluating the HiBalance (See above) program (the BETA-PD study) on PA. A further aim was to examine factors associated with a training effect on PA. 83 participants (Training *n* = 43; Control *n* = 40). Described as ‘highly challenging’ but intensity not reported.
**Penko et al. (2021). (15) USA**	Effect of Aerobic Exercise on Cardiopulmonary Responses and Predictors of Change in Individuals With PD.	Single-center, parallel-group, rater-blind study to determine the effect of aerobic exercise on maximal and submaximal cardiopulmonary responses and predictors of change in individuals with PD. Moderate to high intensity. Participants were enrolled in a trial evaluating the effect of cycling on PD and randomized to either voluntary exercise (VE) (*n* = 35), forced exercise (FE) (*n* = 35), or a no exercise control group (*n* = 20). The exercise groups were time and intensity matched. All exercise was performed 3 times per week for 8 weeks and consisted of a 5–10 min warm-up of light intensity, 40 min of exercise within 60–80% heart rate reserve, and a 5–10 min cool-down of lower intensity.
**Rafferty et al. (2019) (16) USA**	Using Implementation Frameworks to Provide Proactive Physical Therapy for People With PD.	A case report. Role of the physical therapist in 4 paths. High intensity in path 2. 4 paths: (1) A 1-visit consultation path leading to discharge with recommended long-term follow-up re-evaluation, (2) a 2 to 4 visit exercise instruction path, (3) a skilled maintenance path, or (4) a traditional restorative path. 14 (50.0%) individuals were discharged after 1 consultative evaluation and exercise prescription session, 10 individuals (36.0%) had 2 to 4 total visits focused on exercise instruction, 1 had 7 visits with a skilled maintenance delivery pattern over 10.5 months, and 3 completed bouts of restorative PT with 13 to 18 visits. The average duration of the initial episode of care was 8.8 (4.7) weeks.
**Schenkman et al. (2012). (17) USA**	Exercise for People in Early- or Mid-Stage PD.	A 16-Month RCT. The purpose of the study was to compare short- and long-term responses among 2 supervised exercise programs.Moderate intensity in Aerobic Exercise (AE) group.(1. AE training (*n* = 31) 2. Flexibility/Balance/Functional (FBF) training (*n* = 33) and a home-based control exercise program (*n* = 32). All participants were encouraged to perform their prescribed exercise program a total of 5 to 7 days a week throughout the 16 months. AE groups participated in supervised exercise 3 days a week for 4 months. In month 5, supervision was tapered. Thereafter, participants were asked to participate in a supervised exercise session once a month.
**Schenkman et al. (2018). (18) USA**	Effect of High-Intensity Treadmill Exercise on Motor Symptoms in Patients with De Novo Parkinson Disease.	A Phase 2 RCT. To examine the feasibility and safety of high-intensity treadmill exercise in patients with de novo Parkinson disease who are not taking medication and whether the effect on motor symptoms warrants a phase 3 trial. High and moderate intensity interventions. High-intensity treadmill exercise (4 days per week, 80–85%maximum heart rate (*n* = 43)), moderate-intensity treadmill exercise (4 days per week, 60–65% maximum heart rate (*n* = 45)), or wait-list control (*n* = 40) for 6 months.
**So et al. (2023). (19) South Korea**	Home-Based Self-Management Intervention for Community-Dwelling Patients with Early PD.	Feasibility RCT using a pretest-posttest design. Home-based self-management intervention: face-to-face education, telephone counselling and coaching, smartphone-based text messages and information, and smart wearable devices for exercise (*n* = 15). Control group (*n* = 17) 1, 10–15 min session ‘education for PD.’Exercise intensity monitored through participant self-report questionnaire (IPAQ-S). Intervention 14 weeks duration (telephone counselling and coaching weeks 2, 4, 6, 10, and 14).
**Solla et al. (2019). (20) Italy**	Evaluation of the effects of Sardinian folk dance (Ballu Sardu) on functional performance in individuals with PD.	Single-blind, randomized controlled pilot trial. Consecutive sampling approach. The exercise group (*n* = 10) received usual care (medical therapy) plus a 12-week Ballu Sardu dance program, while individuals in the control group (*n* = 9) maintained their habitual activities, and continued their usual care involving medical therapy alone. Intervention intensity not stated. The dance program consisted of two sessions/week, 90-min/class, for 12 weeks.
**Tucak et al. (2023). (21) Australia**	Investigation of the short-term functional and QoL outcomes after the PD-Warrior exercise program.	Single-site, prospective, single-arm pilot study investigating outcomes after the PD-Warrior program for 20 (17 completed) individuals with early PD. High intensity (Intensity of exercise was based on a modification of a commonly used rating of perceived exertion (RPE) scale and is a self-recorded percentage measure of effort, with the goal being 80%. This was used both in the initial assessment sessions and during the classes).Weekly 1 h circuit class (5 min warm-up, followed by 16 stations, each of 2 min of exercises followed by a team game for 5 min, stretches for 5 min and an education period) and completion of a daily tailored home exercise program for 10 weeks.
**Vistven et al. (2023). (22) Norway**	Experiences of an intensive interdisciplinary rehabilitation for people with early-stage Parkinson’s disease	Qualitative, semi-structured interviews using thematic analysis of 7 individuals with early PD researching experiences of an intensive interdisciplinary program and their transition back to everyday life following rehabilitation. Interdisciplinary rehabilitation program organized by Norway’s specialist public health services. Inspired by evidence-based knowledge and the European guidelines, the program emphasized self-management. Promoted behavior change relating to knowledge; concern; competence; and self-esteem.The specialized health care clinic offered individually tailored rehabilitation on an in-patient or day-patient basis. The in-patient rehabilitation program lasted between 3 and 5 weeks, but for day patients extended over 6 weeks, with patients checking in 2–3 times a week. 3 different research-based (High-intensity) exercise approaches: PWR-moves; Rock Steady Boxing; and LSVT Loud. In addition, patients had the choice of participating in various self-efficacy activities, including outdoor climbing, hiking, golf, training in the pool, badminton and table tennis.

*BETA*-*PD* Balance, Elderly Training and Activity in Parkinson’s Disease, *IPAQ*-*S* International Physical Activity Questionnaire Short Form; *LSVT* Lee Silverman Voice Technique, *min*(s) minute(s), *MVPA* moderate to vigorous physical activity, *n* number, *NHS* National Health Service, *PA* physical activity, *PD* Parkinson’s disease, *PWR* Parkinson’s Wellness Recovery, *QoL* quality of life, *RCT* randomized controlled trial

**Table 2:** Outcomes, findings and recommendations of the included studies

Author, date and country	Outcomes relating to PA	Findings	Recommendations
**Bang and Shin (2017). (1) Republic of Korea**	6 MWT	The NWT group exhibited greater improvement in the 6MWT (*p* = 0.003; 95% CI 20.302 to 42.097) compared to the TT group.	Nordic walking could be incorporated into exercise programs of patients with PD to improve balance and walking ability.
**Cancela-Carral et al. (2022). (2) Spain**	The Senior Fitness Test is a battery of tests for the assessment of the physical and functional fitness of PD patients. Provides global score (ΣFitness)	Men showed a trend toward a deterioration in this parameter over the 8 years of follow-up (ΣFitness = − 1.82%, sig = 0.930), while women showed a trend towards improvement (ΣFitness = 0.96%, sig = 0.821).	Regular and systematized practice of long-term physical exercise (regardless of type) helps to slow down the physical deterioration of physical condition in people with PD.
**Carda et al. (2012). (3) Switzerland**	6 MWT	At baseline, the 2 groups did not differ. At the 6-month follow-up, both groups had improved significantly in the primary outcome measure (Lokomat: 458.6 m, 95% CI = 417.23–499.96, *p* =.01; treadmill: mean = 490.95 m, 95% CI = 448.56–533.34, *p* =.0006), but no significant differences were found between the 2 groups (*p* =.53).	Robotic gait training with the Lokomat is not superior to treadmill training in improving gait performance in patients with PD. Both approaches are safe, with results maintained for up to 6 months. The strengths and limitations of robotic devices such as the Lokomat should also be evaluated in terms of costs in future larger multicenter trials.
**Clarke et al. (2016). (4) UK**	Parkinson’s Disease Questionnaire-39 (PDQ-39)	A small but significant difference in the summary index of the PDQ-39 between therapy and control groups (curves diverging at 1.6 points per annum, 95% CI 0.47 to 2.62; *p* = 0.005) but an absence of any motor effect in the PDQ-39 mobility domain NHS physiotherapy and occupational therapy did not produce short or long term clinically meaningful improvements in ADL in patients with mild to moderate PD.	More formalized and intensive physical therapy programs should be developed for different stages of PD. These should then be tested in large-scale RCTs at all stages of PD.
**Ellis et al. (2019). (5) USA**	Mean change in daily steps; mean change in moderate intensity minutes; PDQ-39; 6MWT	The change in 6MWT from baseline to 1 year (33.8 m, 95% CI 5.1 to 62.5) was statistically significant (*P* =.02) and could be considered clinically meaningful for the mHealth group but not the Active control group (5.3 m, 95% CI − 25.6 to 36.2). Both groups improved PA compared with expected activity decline over 1 year. The addition of the mHealth app to exercise intervention appeared to differentially benefit the more sedentary participants.	Further study in a larger group of people with low activity at baseline recommended
**Fishel et al. (2020). (6) USA**	6MWT, PDQ-39 measured at Baseline, 4, 8 and 20 weeks	Participant 3 showed improvement in 6MWT at 4 week follow-up (+ 83 m) beyond minimal detectable change but back to near baseline by 20 week follow-up (+ 8 m). No change in PDQ-39 beyond minimal detectable change.	Additional randomized trials needed to determine how the LSVT BIG intervention may compare to other types of therapy programs for people with PD. Strategies to reduce barriers to exercise and improve HEP long-term compliance will assist therapists and patients in maximizing the benefits of the program.
**Frazzitta et al. (2015). (7) Italy**	6MWT	While no significant changes were found over time in the control group, all variables significantly improved over time in the MIRT Group apart from 6MWT. The distance walked by patients in the 6MWT (around 400 m) corresponds to an average speed of 4 km/h, which is in the range of normal people of comparable age. Therefore, 6MWT does not appear to be a sensitive marker in the earliest stages of the disease. Other results (UPDRS, TUG, PDDS) suggest that MIRT might slow down the progression of motor decay and may have a neuroprotective effect.	Results suggest that MIRT, in the early stage of disease, can not only slow down disease progression, but it can also lead to a better motor performance. This conclusion is also in agreement with the current guidelines, which encourage patients with PD to begin exercise training programs with a high training intensity.
**Handlery et al. (2021). (8) USA**	Daily steps; MVPA; VO_2_ peak	Both exercise groups met targeted treadmill exercise intensity. Neither number of exercise sessions or average steps statistically differed between the 2 exercise groups. Significant increases in daily steps at 5 and/or 6 months for the high intensity group (median of differences = 1250 steps, z = −2.35, *p* =.02). No statistically significant changes for the moderate-intensity group (median of differences = 361 steps, z = − 1.15, *p* =.25) or waitlist control group (median of differences = − 259 steps, z = − 0.04, *p* =.97). Changes in average daily steps: within-group effect sizes (r) 0.44, 0.33, and 0.01 for high-intensity, moderate-intensity, and waitlist control groups, respectively. Daily MVPA: High-intensity group demonstrated a significant increase in daily MVPA (median of differences = 12.5 min, z = −2.67, *p* =.01). Moderate-intensity and waitlist control groups did not show significant changes (median of differences = 6.8 min, z = −1.15, *p* = 0.25; and median of differences = − 0.9 min, z = − 0.04, *p* =.97, respectively). Within-group effect sizes were 0.50, 0.33, and 0.01 for the high-intensity, moderate- intensity, and waitlist control groups, respectively. Changes in VO_2_ peak: exercise groups: no significant within-group changes (median of differences = 0.8 mL/min/kg, z = − 1.85, *p* =.06; and median of differences = −0.4 mL/min/kg, z = − 0.31, *p* =.75, respectively). Control group had a significant decrease (median of differences = − 1.1 mL/min/kg, z = −2.04, *p* =.04) at 6 months, equating to a 5% reduction from the baseline. Within-group effect sizes were 0.36, 0.09, and − 0.42 for the high-intensity, moderate-intensity, and waitlist control groups, respectively. Changes in daily steps were not significantly associated with changes in VO_2_ peak (r = 0.183, *p* =.16).	While the true value of daily step counts is still being determined, the present study provides new information on the relationship between steps and health and provides evidence supporting exercise as a tool to increase PA in people with de novo PD. Additional work is needed to determine the best means of maintaining and continuing to improve both PA and health in people with PD.
**van der Kolk et al. (2019). (9) Netherlands**	6MWT; PDQ-39; Cardio vascular fitness (VO₂ max with graded maximal exercise testing)	Physical fitness improved in the aerobic exercise group (within-group change in VO₂ max 2.0 mL/kg per min), whereas it decreased in controls (− 0.4 mL/kg per min), resulting in a between-group adjusted mean difference of 2.4 mL/kg per min (95% CI 1.1 to 3.7). All other secondary outcomes showed no between-group differences. Park-in-Shape has good potential for a wider implementation with good long-term adherence.	Aerobic home-based exercise is a valuable non-pharmacological treatment for patients with PD with mild disease severity.
**Landers et al. (2019). (10) USA**	Attaining CDC exercise guidelines; IPAQ; 6MWT; PDQ-39	Intervention group had statistically significant improvements: Attaining CDC guidelines (*p* = 0.013); IPAQ moderate physical activity (*p* = 0.004); 6MWT on medication (*p* = 0.017). Control group had statistically significant improvements in: IPAQ vigorous activity (*p* = 0.026), 6MWT on medication (*p* = 0.015).	The high-intensity multimodal exercise boot camp protocol is feasible for people with PD with good compliance and better attainment of CDC aerobic and strength guidelines than usual care. Results suggest that exercise intensity may matter as those in the intervention arm had more improvements across more domains than control.
**Leavy et al. (2019). (11) Sweden**	Qualitative Content Analysis	Themes emerged of ‘applying balance training in daily life’; ‘lack of rehabilitation advice at earlier disease stages’; ‘aware not afraid’; ‘fear and avoidance’; ‘a means to maintain independence.’	People with PD require early advice about the positive effects of PA as well as strategies for self-management.
**Li et al. (2020). (12) Australia**	Exercise behavior (self-reported)	Participants who exercised to recommended levels (150 min per week MVPA) increased from 16% (*n* = 24) at baseline to 44% (*n* = 60) at 1-year follow-up (*p* < 0.001).	The PD Wellbeing Program has been shown to be a successful early intervention model for people with Parkinson’s disease. Future process evaluations of community-based early intervention programs are required to determine the long-term sustainability and outcomes of evidence-based behavior change models of care for people with early stage PD, as well as to identify the program components with the greatest impact on sustaining positive behavior change in this population.
**Moriello et al. (2013). (13) USA.**	PDQ-39 at baseline and 24 weeks; aerobic power was measured using the Cooper 12 min run/walk test at baseline and 24 weeks.	PDQ-39 summary scores on the PDQ-39 improved from an initial score of 20.9 to 4.75 after 24 weeks. No changes were noted in aerobic power.	The intense program was deemed an effective dose of exercise for someone with PD and allowed them to continue to participate in work, leisure, and community activities.
**Nero et al. (2016). (14) Sweden**	Outcome variables were total PA represented by Total Activity Count (TAC), minutes of brisk walking, bouts of brisk walking, sedentary time and sedentary bouts.	The HiBalance program led to an increased amount of brisk walking in daily living but this was not linked to improved balance control. There was a seasonal effect on ambulatory activity in this population, and that the intervention effect dissipated after 6 months.	Recurrent training was recommended to maintain longer-term benefits.
**Penko et al. (2021). (15) USA.**	V0_2_ peak (mL/kg/min) and V0_2_ at Ventilatory Threshold	No significant difference was present for change in V0_2_ peak post intervention, even though the Forced Exercise group had a 5% increase. Both the Forced Exercise and Voluntary Exercise groups had significantly higher percentage oxygen consumption per unit time (VO_2_) at ventilatory threshold (VT) than the control group compared with baseline values (*p* =.04). Mean VO_2_ at VT was 5% (95%CI, 0.1–11%) higher in the FE group (*p* =.04) and 7% (95%CI 2–12%) (*p* =.006) compared with controls.	Peak and submaximal cardiopulmonary function may improve after aerobic exercise in individuals with PD. The improvements observed in aerobic capacity were gained after a relatively short aerobic cycling intervention (i.e., 8 weeks). Further research evaluating the effects of aerobic exercise on PD continues to emerge, which could lead to disease-specific recommendations to mitigate the effect of PD.
**Rafferty et al. (2019). (16) USA.**	6MWT, Change in overall time spent exercising on phone interview 6–12 months after intervention	6 minute walk test at initial evaluation (*n* = 25) 535metres (SD 71); First episode of care (*n* = 5) 582 m (SD 65); Follow-up episode of care (*n* = 4) 641metres (SD 29). Change in overall time spent exercising on phone interview 6–12 months after intervention (*n* = 20): More *n* = 14 (50%); Same *n* = 5 (18%); Less *n* = 1 (3%). Variation in rehabilitation pathway and duration of intervention has potential to impact on results.	Future prospective, controlled trials of PAPT should be completed in early Parkinson’s.
**Schenkman et al. (2012). (17) USA**	Walking economy (oxygen uptake [ml/kg/min]); PDQ-39	Walking economy improved in the AE group compared with the FBF group at 4 months (mean difference 1.2, 95% CI 1.9 to 0.5), 10 months (mean difference 1.2, 95% CI 1.9 to 0.5), and 16 months (mean difference 1.7, 95% CI 2.5 to 1.0). No significant difference in PDQ-39.	PD-specific and endurance programs confer different benefits, with the endurance program having the greatest long-term benefits. Clinicians can extrapolate from these results to determine appropriate exercise programs for individual patients.
**Schenkman et al. (2018). (18) USA**	Exercise frequency: VO₂ max; daily step count by accelerometry	Exercise rates were 2.8 (95% CI 2.4 to 3.2) days per week at 80.2% (95% CI 78.8% to 81.7%) maximum heart rate in the high-intensity group and 3.2 (95% CI 2.8 to 3.6; *p* =.13) days per week at 65.9% (95% CI 64.2% to 67.7%) maximum heart rate in the moderate-intensity group (*p* <.001). VO₂ max improved for participants in the high-intensity group (Mean (SD)) (1.9ml/min/kg (2.9) and decreased in the usual care group (− 1.3 mL/min/kg (2.5)) during 6 months. Change in total step count revealed no difference between groups.	High-intensity treadmill exercise may be feasible and prescribed safely for patients with PD. An efficacy trial is warranted to determine whether high-intensity treadmill exercise produces meaningful clinical benefits in de novo PD.
**So et al. (2023). (19) South Korea**	IPAQ-S; PDQ-39 (Korean version)	The IPAQ-S physical activity score of the intervention group improved significantly more than that of the control group in the intention to treat (Z = − 2.27, *p* =.023) and per-protocol (Z = − 2.80, *p* =.004) analyses.	Home-based intervention (comprising face-to-face education, telephone counselling and coaching, smartphone-based text message and information, and smart wearable devices for exercise) was feasible for patients with early PD. Additionally, the home-based self-management intervention effectively improved physical activity for patients with early PD. Study team suggest further research using a larger sample size.
**Solla et al. (2019). (20) Italy**	6MWT	Post hoc testing with Bonferroni-corrected pairwise comparisons revealed that after the intervention, there was a statistically significant 72.4% increase in the distance participants in the Ballu Sardu group were able to cover during the 6MWT, with a large between-group effect size (ES) (*F* = 41.124; *p* < 0.001; ES 2.98).	Results of this study indicate Ballu Sardu as a safe and feasible form of physical exercise that is likely to have positive effects on functioning in people with PD. In future studies, Ballu Sardu dance may be compared with established exercise training programs and other dance-based activities.
**Tucak et al. (2023). (21) Australia**	6MWT; PDQ-39	Significant improvements were observed in 6MWT distance (mean 484.6 m (SD 75) to 521.8 (SD 64.5) (*p* < 0.001, Cohen’s *D* = 1.16); and the PDQ-39 emotional well-being subdomain (*p* = 0.009; 4.2 points). There was no significant change in any of the other QoL subdomains.	Further studies, ideally in a randomized setting, are needed to confirm these findings and to determine if longer-term participation in the PD Warrior program can slow the progression of the motor and non-motor manifestations of PD.
**Vistven et al. (2023). (22) Norway.**	Qualitative thematic analysis.	Analysis of the data yielded three core themes: being oneself during rehabilitation; believing in oneself again; and managing one’s everyday life following rehabilitation.	Study team report that: Results demonstrate how an intensive interdisciplinary rehabilitation program can contribute to improved physical function and self-esteem for individuals in an early phase of PD. With focus on what they could do, rather than what they could not do, participants developed a sense of capability and mastery. This research points to the need to involve relatives more systematically during the rehabilitation process.

*ADL* activities of daily living, *AE* aerobic exercise, *CDC* Centers for Disease Control and Prevention, *CI* confidence interval, *FBF* flexibility/balance/functional training, *HEP* home exercise plan, *Km*/*h* kilometers per hour, *IPAQ* (-*S*) International Physical Activity Questionnaire (Short-form), *LSVT* Lee Silverman Voice Technique, *max* maximum, *ml*/*kg per min* milliliter per kilogram per minute, *min* minute, *n* number, *MIRT* multidisciplinary intensive rehabilitation treatment, *MVPA* moderate to vigorous physical activity, *NHS* National Health Service, *NWT* Nordic Walking Group, *PA* physical activity, *PD* Parkinson’s disease, *PDDS* Parkinson’s Disease Disability Scale, *PDQ*-*39* 39 Item Parkinson’s Disease Questionnaire, *RCT* randomized controlled trial, *SD* standard deviation, *sig* significance, *TT* time trial only group, *TUG* timed up and go, *UPDRS* Unified Parkinson’s Disease Rating Scale, *VO*₂ (ml/min/kg) volume of oxygen in milliliters per minute per kilogram, *6MWT* 6 minute walk test

### Interventions

The interventions used in the promotion of physical activity in people with newly diagnosed Parkinson’s were varied. Bang and Shin compared an intensive Nordic walking intervention to treadmill walking alone [22]. Schenkman et al. (2018) examined a treadmill training intervention describing it as high intensity treadmill training [42]. Handlery et al. (2021) compared high-intensity and low-intensity treadmill training and Carda et al. (2012) compared body-weight supported to standard treadmill gait training [43, 44]. Other interventions included aquatic therapy [45], cycling [46], dance [47], and yoga [48]. Many studies referred to using aerobic exercise [22, 42, 43, 45, 46, 49–53]. Three studies used technology to support adherence: Ellis et al. used a health ‘app’ supported with provision of a tablet [49]; So et al. (2023) used smartphone-based messages and information alongside smart wearable devices for exercise [54]; van der Kolk et al. utilized exergaming with virtual reality software linked to a stationary home exercise bike [51].

Leavy et al. and Nero et al. focused on the effects of the HiBalance balance intervention, the former using qualitative content analysis with an identifiable cohort of eight people within 5 years of Parkinson’s diagnosis. Other studies had significant elements of balance work within their studies [48, 52, 53, 55, 56]. The Lee Silverman Voice Technique BIG program promotes strategies for control of upper and lower limbs, balance, and gait [55]. Landers et al. (2019) describe high-intensity multi-modal training which includes balance work [53]. Moriello et al. (2013) used a single case study incorporating yoga into an existing intensive physiotherapy program with strength, balance, and agility work [48]. Schenkman et al. (2012) compared aerobic exercise training with flexibility, balance and functional training, and a home exercise control group [52]. The ‘PD Warrior’ program of Tucak et al. (2023) incorporates 10 core exercises, each involving dynamic balance elements [56].

The duration of the interventions also varied widely. They ranged from 4 weeks for the Bang and Shin, Carda et al., and Fishel et al. interventions [22, 44, 55] to 16 months for the Schenkman et al. (2012)intervention [52], and Cancela-Carral et al. monitoring the longitudinal effects of participating in physical activity programs over 8 years. Most were between 2 and 6 months duration [42, 43, 46–48, 51, 53, 54, 56–60] but Frazzitta et al. examined the effects of two, 28-day multidisciplinary rehabilitation inpatient stays, 1 year apart [50].

The widest variability of interventions came within studies by Clarke et al. [57], Rafferty et al. [60], and Vistven et al. [61] all provided from existing healthcare clinics. Clarke et al. examined physiotherapy and occupational therapy in NHS settings in the UK [57], with a mean duration of 8 weeks. The median number of therapy sessions was four (range 1–21) with a mean time per session of 58 min and a mean total dose of 263 min (range 38–1198). Rafferty et al. reported an average duration of 8.8 weeks physiotherapy input but participants had four potential and widely differing pathways: 14 (50%) of participants were discharged after one consultative evaluation and exercise prescription session; 10 (36%) participants had two to four visits focused on exercise instruction; one (4%) had seven visits over 10.5 months; and three (10%) participants had between 13 and 18 physiotherapy visits [60]. Vistven et al. offered individually tailored rehabilitation programs for both inpatients or day-patients lasting between 3 and 6 weeks with three different high-intensity approaches (‘Parkinson’s Wellness Recovery,’ ‘Rock Steady Boxing,’ or Lee Silverman Voice Technique) in addition to activities with a social element for self-efficacy including hiking, golf and table-tennis [61].

Less than half of the studies had a consistent dose and duration [42–44, 46, 50, 53, 55, 58, 59]. There were considerable differences in the duration and frequency of the individual sessions across the other studies. Where stated, sessions varied from 30 to 90 min and from twice weekly to five to seven times weekly. Most studies reported moderate or high-intensity interventions [22, 42–44, 46, 48–53, 55, 60]. The methods used to determine intensity are discussed in subsequent sections.

Several authors have highlighted the importance of behavior change strategies but few referred back to specific models. Many studies highlighted the importance of goal setting [48, 49, 55, 60, 62]. Rafferty et al. related goal setting to shared decision making [60]. Li et al. reported the need to incorporate personalized behavior change techniques such as goal-setting and linked this to self-efficacy [62]. Several others highlighted the importance of autonomy and self-efficacy [50, 52, 58, 59], but only Schenkman et al. (2012) specifically highlighted Bandura’s self-efficacy theory [63]. Van der Kolk et al. highlighted the potential benefits of their motivational app [51]; Ellis et al. described how their app allowed behavioral change elements to be embedded in home exercise prescription from basic (goal-setting tailored instruction, feedback) to more advanced (ongoing goal-setting and feedback, mastery experiences, self-reflection, and more extended support from a health care professional) [49]. Nero et al. proposed an ecological model describing determinants of PA including intra-, and interpersonal, as well as environmental factors [59].

### Population

As specified in the inclusion criteria, all studies involved people within 5 years of Parkinson’s diagnosis. Most studies had an average participant age of over 60 years [42–47, 49, 50, 52, 53, 55–62], reflecting that most people with Parkinson’s have received their diagnosis when they are older than 60 years [3]. Clarke et al. had the broadest age range of participants, from 35 to 91 years [57]. Of the studies with multiple participants, most had majority male participants, ranging from 53 to 94% of the sample [42–47, 49, 51–54, 56, 57, 59, 60, 62].

As might be expected in newly diagnosed Parkinson’s populations, H&Y was most often described as stages 1 to 3, with only Cancela-Carral et al. Clarke et al. and Li et al. including participants with H&Y stages 1 to 4 [45, 57, 62]. Only Moriello et al. and Penko et al. did not report H&Y status but Penko et al. did provide the Unified Parkinson’s Disease Rating Scale (UPDRS) motor sub-score as an alternative indication of disease severity at baseline (See supplementary information) [46, 48].

Sixteen studies reported participant medication usage at study start [42–44, 46–48, 50, 51, 53–57, 59, 60, 62]. Of these, only one study used participants who were entirely Parkinson’s medication naïve [42] with three other studies with identifiable participants/cohorts as levodopa naïve [43, 50, 55]. Eleven studies gave reported dosage [44, 46–48, 51, 53, 54, 56, 57, 59, 62] with ten of these reporting Levodopa dose equivalency (LDE) [44, 46, 47, 51, 53, 54, 56, 57, 59, 62].

Other participant characteristic reporting differed between studies. Li et al. provided the most detail: in addition to employment status and preferred language, they reported baseline comorbidities, exercise status, Mini-Mental State Examination (MMSE) score, history of falls in the last year, Freezing of Gait (FOG), Berg balance score, Parkinson’s Fatigue Scale and Depression, Anxiety and Stress Scale scores [62]. Only four other studies reported on cognitive screening at baseline, despite cognitive impairment being a significant non-motor symptom for many pwp with the potential to impact on study participation [1]: three used the MMSE [22, 44, 58]; Schenkman et al. (2018) used the Montreal Cognitive Assessment Score (MOCA) at baseline [42]. Where reported, all participants were above the threshold for cognitive impairment, as determined by the chosen outcome measure.

Only Cancela-Carral et al., Ellis et al., and Schenkman et al. (2018) provided detailed reporting on participant race, with 100, 91, and 100% white participants respectively [42, 45, 49]. Some studies reported on factors with potential to impact on recruitment bias such as education level [45, 49, 51, 54], employment status [48, 51, 52, 55, 60, 61], annual income [52, 54], or private health insurance status [60]. Again, these details were not consistently reported across the studies. Where reported, participants were educated, employed or retired, and with private health insurance (See supplementary information).

Participant adherence was reported in 95.5% (21/22) of the studies with only Rafferty et al. not reporting adherence [60]. Some reported completion of all face-to-face sessions [22, 28, 64]. Other reported figures for completion of supervised sessions varied between 79 and 97% [65, 66, 67]. Home exercise adherence varied from the Fishel et al. participant reporting 80% completion [55] to Schenkman et al. (2012), who stated that no meaningful data could be gained from home exercise diary [52]. Ellis et al. used exercise diary completion for the active control group but monitoring via the health app for the intervention group. They found similar levels of home exercise adherence between groups with 3.5 days per week on home exercises in the control group and 3.2 days per week on exercise in the intervention group [49]. Handlery et al. used National Health and Nutrition Examination Survey (NHANES) rules [68] to define valid accelerometer wear time as those with more than 10 h wear time each day over a 10-day period and reported mean wear time of more than 13 h per day [43]. Nero et al. attempted to monitor participant adherence using accelerometer data but, of the 100 included randomized participants, 76 completed 12-month follow up. Of these, 66 contributed physical activity data due to invalid or missing accelerometer data, suggesting technical as well as adherence issues [59].

### Characteristics of professionals

 Most studies utilized interventions within the scope of practice of physiotherapists and seventeen studies included delivery by physiotherapists. Only Penko et al. and So et al.described interventions exclusively delivered by other professions with exercise physiologists and community health nurses respectively [46, 54]. Schenkman et al. stated that the intervention was delivered by study coordinators but their professional background was not clear [42]. Solla et al. and Tucak et al. delivered interventions from those with a specialist exercise instructor qualification [47, 56]. Clarke et al.described physiotherapy and occupational therapy interventions [57]. Frazzitta et al. and Li et al. described multidisciplinary rehabilitation [50, 62].

 Study team fidelity was not reported in eight studies [22, 45, 47, 48, 50, 51, 54, 55]. Where reported, fidelity techniques varied from selecting from predetermined programs [47, 49, 53, 56, 59], to co-treatment with the primary investigator [52, 60], and regular study team conference calls [42, 43].

### Settings

 The USA was the largest provider of interventions [42, 43, 46, 48, 49, 52, 53, 55, 60]. Two studies were delivered in Australia [56, 62], two in the Republic of Korea [22, 54], and the remaining in Europe, including the UK [57], Italy [47, 50], the Netherlands [51], Norway [61], Sweden [58, 59], Switzerland [44], and Spain [45].

The majority of the studies provided interventions within outpatient clinics [42, 43, 47, 48, 52, 54–57, 60–62]. In the Bang and Shin study, recruitment took place in a University Medical Centre but it was unclear whether the intervention was provided in the same clinic setting [22]. One study provided interventions either in an inpatient or an outpatient setting [61]. Only one study was conducted exclusively on a ward setting [50]. Several of the interventions provided in clinical settings also explicitly involved continuation of interventions in free-living environments [48, 52, 60]. Four of the interventions were solely conducted in free-living environments [49, 51, 58, 59].

### Methodology and outcome measures

 Fourteen studies were described as randomized trials [22, 42–44, 46, 47, 49–54, 57, 59]. Of these, four were described as single-blind [22, 44, 46, 47], two described as feasibility [53, 54], and three described as pilot studies [47, 49, 50]. Tucak et al. described their study as a pilot but this was non-randomized, single-site, and prospective [56]. By far the largest controlled trial was conducted by Clarke et al. with 762 participants [57]. The other randomized trials had between 19 and 130 participants [22, 42–44, 46, 47, 49–54, 59] with nine of these having 35 participants or less in each arm [22, 44, 46, 47, 49, 50, 52–54]. Three articles did not refer to sample size calculations to justify their sample size [22, 43, 46]. There were two retrospective cohort studies with 71 and 152 participants at baseline respectively [45, 62]. Additionally, there was one case series with three participants [55] and two case reports, one with a single case [48] and one with 28 participants that were unevenly distributed between interventions [60]. Only two studies used qualitative research methodology with semi-structured interviews: one using qualitative content analysis to capture eight participant views on the HiBalance intervention [58], and one using thematic analysis to explore seven participant views of a three-week interdisciplinary rehabilitation program [61].

 A wide variety of outcome measures were used across the studies but this scoping review focused on those that could be used to indicate a change in activity levels or, by proxy, a change in fitness levels (including qualitative participant feedback relating to these aims). Only eight studies enabled the calculation of physical activity accrued at moderate and above intensity physical activity levels [42, 43, 49, 52–54, 60, 62]. Those that did, did this in very different ways. Ellis et al. equated moderate intensity minutes to number of minutes with more than 100 steps per minute (ankle-worn accelerometer) [49]. Handlery et al. took 100 steps per minute as the threshold for light intensity with a much higher threshold of 1952 steps per minute as the threshold for moderate intensity physical activity (wrist-worn accelerometer) [43]. Landers et al. used the International Physical Activity Questionnaire (IPAQ) as well as indicative heart rate during clinic sessions [53]. So et al. used the IPAQ short form questionnaire (IPAQ-S). Li et al. and Rafferty et al. used more basic self-report in the form of a questionnaire and phone survey respectively to determine those reporting more than 150 min moderate and above physical activity [60, 62]. Rafferty et al. only captured self-report in 20 out of 28 participants [60]. Schenkman et al. used minutes at indicative heart rates for moderate or vigorous activity [42, 52]. Other methods used that could potentially be used to calculate intensity of physical activity were rate of perceived exertion [55] and minutes of brisk walking (equating step counts from a waist-worn accelerometer to a walking speed threshold for brisk walking) [59].

 The six minute walk (6MWT) test was the most frequently used clinician/researcher-reported outcome measure in nine of the studies [22, 44, 47, 49–51, 55, 56, 60]. The 6MWT has been shown to be a useful measure of aerobic capacity in patients with chronic disease due to its correlation with maximal oxygen uptake (VO_2_ max) [69]. The Cooper 12 min run/walk test was used by Moriello et al. as an alternative measure of aerobic capacity, which is also predictive of VO_2_ max [48].

 Van der Kolk et al. used the VO_2_ max in conjunction with the 6MWT and 39-item Parkinson’s Disease Questionnaire (PDQ-39) [51]. Handlery et al.used VO_2_ max (described as VO_2_ peak) alongside steps per minute. Schenkman et al. (2012) focused on walking economy, determining volume of oxygen uptake (VO_2_) at different walking speeds, rather than a maximal exercise test [52]. Penko et al. stated that VO_2_may be underrepresenting any cardiorespiratory improvements gained as a result of an exercise intervention if VO_2_ peak is blunted in individuals with PD due to chronotropic incompetence. They therefore used VO_2_ peak at ventilatory threshold as a submaximal index of aerobic capacity, positing this as a more relevant and meaningful change in aerobic capacity [46].

 Schenkman et al. (2018) used VO_2_ max and heart rate in conjunction with daily step count using a tri-axial accelerometer (Actigraph GT3X) to determine the volume and frequency of exercise [42]. Nero et al. also used the Actigraph GT3X accelerometer to measure daily step count and minutes of brisk walking derived from a separate study on pwp within the same thesis. This was not explicitly linked to levels of moderate and above levels of physical activity [59]. Reflecting the wide range of approaches in accelerometer physical activity research, Ellis et al. chose to use a uniaxial accelerometer using step count to determine mean change in daily steps and mean change in moderate and above intensity minutes of physical activity [49]. In these studies, wearable physical activity monitors were used as passive monitoring devices rather than for self-monitoring and feedback to support behavior change techniques to increase physical activity [70].

 The most commonly used patient reported outcome measure relevant to physical activity was the PDQ-39. The PDQ-39 contains several items, which provide an indication of difficulty with leisure activities and activity participation and so was included as a relevant outcome measure in this scoping review. The PDQ-39 was the only physical activity- relevant outcome measure included in the Clarke et al. study [57]. More often, the PDQ-39 was reported alongside other objective measures. It was included alongside the 6MWT in four of the studies [49, 51, 55, 56]. Other objective measures used alongside the PDQ-39 included the Cooper 12 min walk test used by Moriello et al. [48] and walking economy used by Schenkman et al. (2012) [52].

### Key findings and recommendations from study authors

 Bang and Shin used the improvement in 6MWT (see Table 2) in the Nordic Walking Group to assert that Nordic walking could be incorporated into the exercise program of patients with Parkinson’s to improve balance and walking ability [22] Ellis et al. found a statistically significant change in 6MWT from baseline to 1 year that was considered clinically meaningful for the mHealth group but not the active control group. They found that both active and control groups improved physical activity in terms of total steps per day or minutes containing 100 or more steps per minute (Their defined threshold for moderate and above intensity physical activity), although this change was more pronounced in the participants with low activity at baseline. The change in 6MWT was used as evidence to support the mHealth app as an intervention to support physical activity in pwp but they recommended further study in a larger group of people with low activity at baseline [49].

 Rafferty et al. also used improvements in 6MWT in support of the proactive physical therapy (PAPT) program but this was only captured in four participants at follow-up episode of care. Subjective self-reported improvements in time spent exercising was also used in support of PAPT but, as described above, this was captured in part of the sample (20 out of 28), with variation in rehabilitation pathway and duration of intervention potentially impacting on results. Future controlled trials of PAPT were therefore recommended in early Parkinson’s [60].

 In their small trial, Solla et al. found significant increases in the 6MWT for their Sardininan Bella Sardu folk dance 12-week intervention, recommending future studies comparing Bella Sardu to other more established exercise interventions [47].

 In their case study, Fishel et al. also found an improvement in 6MWT beyond the minimal detectable change at 4 week follow up, but that this was back to baseline by 20-week follow up. They found no change in PDQ-39 beyond minimal detectible change and concluded that additional randomized trials are needed to compare LSVT BIG training with other interventions, but also to explore strategies to reduce barriers to exercise and improve longer-term compliance [55].

 Tucak et al. found a significant increase in the 6MWT and the emotional sub-domain of the PDQ-39 for their PD Warrior 10-week intervention recommending further studies, ideally in a randomized setting, to confirm these findings [56]. Although not related to physical activity levels or fitness, they also highlighted a significant difference in the Unified Parkinson’s Disease Rating Scale (UPDRS). Despite their small sample-size and non-controlled setting, they described the UPDRS result as demonstrating comparable efficacy to other high-intensity interventions such as the Multidisciplinary Intensive Rehabilitation Treatment (MIRT) group of Frazzitta et al. [50].

 Conversely, Frazzitta et al. found no change in the 6MWT in their intervention group and suggested that this outcome measure is not a sensitive marker in the early stages of Parkinson’s. They suspected a ceiling effect in the 6MWT in early Parkinson’s as the distance walked at baseline and follow-up corresponded to the range of people unaffected by Parkinson’s of a similar age. Other variables, not indicative of physical activity levels or fitness, such as the UPDRS and Parkinson’s Disease Disability Scale (PDDS), did show significant improvement in the Multidisciplinary Intensive Rehabilitation Treatment (MIRT) group and were used in evidence to support MIRT [50].

 Carda et al. found a significant improvement in 6MWT in both the Lokomat and regular treadmill training groups at 6 months follow up with no significant differences between groups. They concluded that robotic gait training with the Lokomat is not superior to treadmill training in improving gait performance in pwp and that the strengths and limitations of robotic devices such as the Lokomat should be evaluated in terms of costs in future larger multicenter trials [44].

 Van der Kolk et al. also found no between-group difference with the 6MWT and also the PDQ-39 [51]. However, physical fitness, as defined by changes in VO_2_ max, improved in their aerobic exercise group and decreased in the controls. The results at 6 months in this study were used to support the potential for the Park-in-Shape home-based approach to aid long-term physical activity adherence for pwp with mild disease severity. Further studies with larger sample sizes were recommended.

 Penko et al. used VO_2_ peak at ventilatory threshold as an alternative to VO_2_ max. They found that forced exercise and voluntary exercise groups engaging in a semi-recumbent stationary cycle exercise program had significantly higher percentage oxygen consumption per unit time (VO_2_) at ventilatory threshold (VT) than the control group compared with baseline values. Penko et al. highlighted that the improvements observed in aerobic capacity were gained after a relatively short aerobic intervention (i.e., 8 weeks) but had no longer-term follow-up [46].

 In their 8-year longitudinal study, using summary scores from a battery of tests for the assessment of the physical and functional fitness of pwp, Cancela-Carral et al. found a slight decline in the summary score for male participants, but an improvement in female participants (see Table 2). Not having a non-exercise control group was highlighted by the authors as a limitation of this study [45].

 In their large randomized controlled trial with 15-month follow-up, Clarke et al. found a small but significant difference in the summary index of the PDQ-39 between therapy and control groups (see Table 2), but no motor effect was observed in the PDQ-39 mobility domain [57]. They concluded that this is likely to be multifactorial because of the early disease stage of most of the patients, the low dose of intervention, and the lack of consistency in therapy assessment and intervention approaches. Overall, they reported that NHS physiotherapy and occupational therapy did not produce short- or long-term clinically meaningful improvements in activities of daily living, and that more formalized and intensive physical therapy programs should be developed and tested in in large-scale randomized controlled trials for all stages of Parkinson’s.

 In their single case study, Moriello et al. found scores on the PDQ-39 improved from an initial score of 20.9 to 4.75 after 24 weeks [48]. Although Moriello et al. did not report on H&Y status, normative data for the PDQ-39 summary score (Mean (Standard Deviation)) at different stages are reported to be: H&Y 1: 18.39 (14.37) *n* = 33; H&Y 2: 31.6 (17) *n* = 56; H&Y 3: 36.53 (19.64), *n* = 20 [71]. Moriello reported no changes in aerobic power as determined by the Cooper 12 min run/walk test but suggested that their intense program was an effective dose of exercise for someone with Parkinson’s, allowing the participant to continue with work, leisure, and community activities.

 Contrary to the findings of Moriello et al. [48], in a larger sample, Schenkman et al. (2012) found no significant difference in PDQ-39, but an improvement in aerobic capacity (Determined by walking economy) for an Aerobic Exercise Group compared to a Flexibility, Balance and Functional exercise group [52]. They also concluded that Parkinson’s specific and endurance programs confer different benefits with aerobic exercise training providing longer-term benefits on functional capacity. Their recommendations were similar to Moriello et al. [48] stating that appropriate exercise programs need to be determined by clinicians for individual patients [52].

 Schenkman et al. in 2018 also found different benefits for different exercise focus [42]. They determined that VO_2_ max improved in the high-intensity treadmill group, stayed similar in the moderate-intensity treadmill exercise group, but decreased in the usual care group after six months (see Table 1). They reported that both treadmill exercise groups met WHO physical activity recommendations [72], although volume of physical activity did not change over the course of the study revealing no difference between groups in total daily step count (As determined by triaxial accelerometer). Schenkman et al. (2018) recommended that high-intensity treadmill exercise is feasible and may be prescribed safely for patients with Parkinson’s but that an efficacy trial is warranted to determine whether high-intensity treadmill exercise produces meaningful clinical benefits in de novo Parkinson’s.

 Using the same triaxial accelerometer as Schenkman et al. (2018) (Actigraph GT3X, Actigraph, Pensacola, FL, USA), Handlery et al. did find a significant increase in daily steps at 5 and/or 6 months for the high intensity group but not the moderate-intensity group or waitlist control group. Using step counts per minute to determine the amount of moderate to vigorous PA (MVPA), they demonstrated a significant increase in daily MVPA in the high-intensity group only. Of the 110 participants with valid PA data at baseline, 84 (76%) already met WHO PA recommendations. Handlery et al. did not see significant within group changes in VO_2_ peak or find that changes in daily steps were associated with changes in VO_2_ peak. They acknowledge that the true value of daily step counts is still being determined and that additional work is needed to determine the best means of maintaining and continuing to improve both PA and health in pwp [43].

 Other authors used different methods to determine MVPA [53, 62]. Li et al. used basic self-report responses to a questionnaire to determine participants who exercised to WHO recommended levels and found an increase 16% (*n* = 24) at baseline to 44% (*n* = 60) at 1-year follow-up from their PD Wellbeing Program. They recommended future process evaluations of intervention programs for early stage Parkinson’s to determine the long-term sustainability and outcomes of evidence-based behavior change models [62].

 Landers et al. used self-report from the IPAQ to determine MVPA found that the high-intensity multimodal exercise intervention group had statistically significant improvements in attaining WHO physical activity guidelines and the 6MWT on medication. They did also see significant improvements in vigorous physical activity and 6MWT on medication in the control group suggesting that exercise intensity may matter as those in the intervention arm had more improvements across more domains than control [53].

 Although So et al. relied on self-report from the IPAQ short-form questionnaire, rather than calculate MVPA, they used an overall physical activity score of MET minutes per week to show that the physical activity score of the intervention group improved significantly more than that of the control group [54].

 In paper III of their thesis, Nero et al. used the Actigraph GT3X accelerometer but, rather than calculate MVPA, they used a variation of overall physical activity (total activity counts) and also the amount of brisk walking in daily living. They found that the Hi-Balance program led to an increased amount of brisk walking in daily living but that this was not linked to improved balance control. Using their method of vertical axis total activity counts and their predetermined threshold for brisk walking activity, they found that there was a seasonal effect on ambulatory activity, which was higher in the spring season. They also found that the HiBalance intervention effects on daily walking dissipated after six months suggesting that recurrent training is required [59].

 Leavy et al. used qualitative content analysis to capture eight participants’ views on the HiBalance intervention [58]. Themes emerged of ‘applying balance training in daily life’; ‘lack of rehabilitation advice at earlier disease stages’; ‘aware not afraid’; ‘fear and avoidance’; ‘a means to maintain independence.’ The recommendation was that pwp require early advice about the positive effects of physical activity as well as strategies for self-management in order to ease the psychological and physical burden of progressive balance impairment.

 Vistven et al. used qualitative thematic analysis on the experiences of seven individuals with early Parkinson’s following intensive interdisciplinary exercise programs. Three core themes were identified: ‘being oneself during rehabilitation’; ‘believing in oneself again’; and ‘managing one’s everyday life following rehabilitation.’ They pointed to how results demonstrated how intensive interdisciplinary exercise programs in early Parkinson’s can contribute to feelings of improved physical function and self-esteem. Vistven et al. also pointed to the need to the need to involve relatives more systematically during the rehabilitation process [61].

## Discussion

 Overall, despite research recommendations and a clinical and policy drive toward the promotion of physical activity in people with newly diagnosed Parkinson’s [3, 73], there is limited research with newly diagnosed cohorts. This may, in part, be due to the longstanding debate over defining early Parkinson’s described above.

### Study design and characteristics

 The majority of studies (64%) were randomized controlled trials, which may reflect the focus of the review being on interventional research. Of the fourteen studies that described themselves as randomized, five were double-blind randomized controlled trials advanced beyond the level of pilot or feasibility study. However, variation in study design and outcomes used would preclude a systematic review and meta-analysis at present.

 The baseline characteristics of the participants were similar in terms of age and sex, with majority male participants in most cohorts. This may reflect that Parkinson’s occurs more commonly in men than women and those over the age of 60 [1, 2], but also the lack of involvement of female participants in research generally, and in Parkinson’s research more specifically [74]. However, other demographic details were lacking in many of the studies. Detailed reporting of participant characteristics, for example, ethnicity, cognitive status, is an important element in research reporting to allow review of whether research participants reflect those who might benefit from the results and are inclusive and representative of the patient population [75].

 Lack of, or different reporting of medication usage at study start made it difficult to determine if this characteristic was similar across all studies. Four studies enabled identification of levodopa naïve participants or cohorts [42, 43, 50, 55]. corresponding to one definition of ‘de novo’ Parkinson’s but changes to prescribing practices have broadened this definition, arguably making it a less useful identifier of early Parkinson’s.

 Studies were predominantly small in sample and none followed recommended Template for Intervention Description and Replication (TIDieR) guidelines for intervention reporting, which would make a systematic review and/or meta-analysis more challenging [76]. Details on aspects such as content and delivery location were not always clear across the included studies.

 The study settings varied from ward and clinic settings, to free-living environments and combinations of both. This may reflect the school of thought that clinical diagnostic measures and disease-progression markers remain sub-optimal for Parkinson’s management and that remote physical activity monitoring may correlate more accurately with Parkinson’s disease specific predictors, outcomes, and interventions [77, 78–80].

 The 6MWT was the most frequently used outcome measure, but there is concern over a possible ceiling effect in this measure for people with newly diagnosed Parkinson’s. The PDQ-39 was the second most common outcome measure, but self-report questionnaires require retrospective recall and can be affected by external factors such as social desirability [81].

 Remote monitoring offers the ability to monitor over longer time periods, potentially providing a more objective view of physical activity levels, but can be expensive and require additional resources to provide/collect [81]. Nero et al. demonstrated the potential for technical issues affecting remote monitoring [59]. There is also debate over the relative advantages and disadvantages of remote monitoring with feedback to participants to promote behavior change, or passive monitoring as a more objective method [70, 82]. This is coupled with concerns over the accuracy of different physical activity monitors, physical activity monitor location, and methods to determine the intensity of physical activity using these monitors in Parkinson’s populations [78, 82, 83]

### Interventions to promote physical activity

 The review demonstrated that the interventions in the identified literature are varied although delivery of interventions was predominantly led by physiotherapists. The difficulties in defining physiotherapy interventions for Parkinson’s are well-documented and often incorporate a broad definition of traditional physiotherapy techniques and multi-faceted interventions [3, 38, 39, 84, 85].

The American College of Sports Medicine states that in addition to defining physical activity, it is important to define the range of intensities associated with physical activity and the different methods used to estimate intensities [86]. The use of steps per minute [43, 49], minutes of brisk walking [59], self-report [53, 54, 60, 62], heart rate [42, 51–53], and rate of perceived exertion [55] as measures of intensity by the different studies shows the range of different methods. Few studies justified their thresholds for moderate- and high-intensity exercise [49, 43, 51, 53, 54]. There was also some variation in the thresholds used, for example, while Schenkman et al. (2018) used a percentage of 60–65% maximum heart rate to define moderate-intensity and 80–85% maximum heart rate for high-intensity exercise, Schenkman et al. (2012) and Van der Kolk et al. used ranges of 50% to 80% as bounds for moderate intensity exercise. No studies used metabolic equivalents (METs) or MET levels to determine exercise intensity. These are a standardized method for quantifying the absolute intensity of physical activity and exercise, and can be useful as relative exercise intensity changes with age-related declines in maximal aerobic capacity [86].

 Three studies justified the use of VO_2_ max, widely considered to be the gold standard measurement of aerobic capacity [42, 43, 52], but there are concerns over its use in patient populations and subjects who are inexperienced or unwilling to push themselves to exhaustion [87]. As described above, Penko et al. highlighted that VO_2_max may be blunted in individuals with Parkinson’s [46]. This measure is also not feasible in community-based studies with non-laboratory-based outcome measures.

 The wide range of methods used to determine physical activity intensity in the studies reflects the range in use in physical activity definitions and recommendations. Most of the studies reported high-intensity interventions [22, 42–44, 46, 48, 50, 51, 53, 55, 56, 60, 61]. This is in keeping with current recommendations for people with newly diagnosed Parkinson’s based on animal and a small number of human studies suggestive of neuroprotective and potentially neurorestorative effects [12, 15, 73, 88]. However, less than half of the studies [42, 43, 49, 52–54, 60, 62] related physical activity outcomes back to WHO guidelines of 150 min of moderate and above physical activity per week [72] or enabled the calculation of physical activity accrued at these levels. The differences in dose and duration reported also made it difficult to determine if participants were achieving WHO recommendations in many of the studies.

 A major obstacle to improving physical activity counselling and designing appropriate interventions is the lack of knowledge of baseline activity habits [89]. The difficulties associated with objective measurement of physical activity levels also supports triangulation of data through mixed-methods approaches. Mixed methods and qualitative approaches are more likely to inform the social determinants of the success or failure of interventions including acceptability. They may also help to determine the best settings for interventions. Consideration and reporting of the behavior change models used in interventions and the active involvement of pwp in data interpretation stands to provide personally and clinically meaningful endpoints to inform advances in translational research for pwp [90].

 It may be that messaging around high-intensity exercise is not always beneficial. Focus group research into activity promotion in general and in Parkinson’s populations have highlighted that the word ‘exercise’ can be a barrier with negative connotations [91, 92]. They recommend the use of the term ‘physical activity’ over ‘exercise’ and WHO highlight the key message that some physical activity is better than none. This demonstrates the importance of the participant voice in research and is in keeping with the Leavy et al. recommendations that pwp require early advice about the positive effects of physical activity as a means to maintain independence [58]. A large meta-analysis of ‘physical activity’ interventions by health professionals for general populations in primary care settings included studies where interventions were delivered or prompted by health professionals and showed a positive effect on participation in moderate to vigorous physical activity participation [93]. This highlights that messaging around moderate and vigorous physical activity and relating advice back to easily understandable guidelines may be key and need further investigation in more specific populations.

### Limitations

 A limitation of this scoping review may be the wide range of terms for exercise and physical activity affecting the search criteria. Initial search terms were developed from keywords from key literature and previous reviews in this area, with further advice from an appropriate University Information Specialist. This does not exclude the possibility that relevant search terms were missed. Further limitations are that studies were limited to those published in English and that the title screening performed by one reviewer only. However, all other screening was performed in line with JBI methods.

 The strict inclusion criteria resulted in a low number of studies to draw conclusions from. However, this reflects the findings from included studies and National Institute for Health and Care Excellence (NICE) guidelines in the UK [20], highlighting the need for further research in newly diagnosed Parkinson’s populations. It may also reflect the general difficulties in research of recruiting a sufficient number of participants and increased difficulties with more specific samples [92]. Many studies that solely used H&Y staging to illustrate stage of Parkinson’s were excluded. While H&Y staging is recognized as being practical to both research and patient care settings, it is an indication of severity rather than duration. There is also criticism that the scale is ordinal and progression across stages is non-linear [25, 26] so this was not considered appropriate to capture newly diagnosed cohorts. A cutoff of 5 years from diagnosis was considered sufficient to capture the research into the newly diagnosed population, but this is subjective. The narrow inclusion criteria may have excluded many studies with conclusions relevant to those newly diagnosed.

## Conclusions

Despite the wide variety in the participant samples, settings, methodology, and outcome measures used, in all but one of the studies [57], the authors interpreted the results as favoring interventions to promote physical activity in people with newly diagnosed Parkinson’s. Due to the acknowledgment of methodological limitations by the authors included in the review, the majority of the included studies recommended further study in this area, including larger controlled trials [42, 44, 49, 51, 54, 56, 57, 60], cost-effectiveness studies [50], and investigation of behavioral factors [55, 59, 62]. Several studies recommended the wider implementation of the interventions in clinical practice [22, 48, 51, 52, 58, 61]. Clarke et al. recommended further investigation in clinical practice involving more formalized and intensive physiotherapy programs for all stages of Parkinson’s [57].

Despite the growing evidence for the value of physical activity and exercise for pwp, the optimal prescription or mode of delivery for different stages of Parkinson’s remains undetermined [73]. Future systematic review and/or meta-analysis are therefore likely to be possible with further developments in this field. The most important behavior change elements to promote physical activity could also be elucidated with qualitative and/or mixed methods approaches to inform the social determinants of intervention success or failure.

### Implications for research

 Further discussion on the definition of newly diagnosed Parkinson’s would be beneficial, as would clearer, more consistent reporting of participant demographics including cognitive status and ethnicity. Corresponding to TIDieR guidelines for intervention reporting [76] would help with interpretation of the results. This would also help to ensure the justification of dose and intensity, and the measures used to define this. It would also help to clarify which specific exercise or physical activity approaches have been utilized and by whom. Relating interventions to WHO guidelines for physical activity [72] may also help with consistent reporting and messaging. The importance of accessibility of intervention settings could also be explored further through Patient and Public Involvement and Engagement (PPIE). Future work could employ a mixed methods approach to fully capture the potential barriers and facilitators that effect intervention acceptability.

## Supplementary information

Supplementary Material 1. Study characteristics.

## Data Availability

Scoping Review registration and protocol available at https://pearl.plymouth.ac.uk/. Search strings available at https://searchrxiv.org/. The data extraction tables used and/or analyzed during the scoping review are available from the corresponding author on reasonable request.

## Appendix A: Preferred Reporting Items for Systematic reviews and Meta-Analyses extension for Scoping Reviews (PRISMA-ScR) Checklist

**Table:**

**Section**	**Item**	**PRISMA-ScR checklist item**	**Reported on page #**
**TITLE**			
Title	1	Identify the report as a scoping review.	1
**ABSTRACT**			
Structured summary	2	Provide a structured summary that includes (as applicable): background, objectives, eligibility criteria, sources of evidence, charting methods, results, and conclusions that relate to the review questions and objectives.	1-2
**INTRODUCTION**			
Rationale	3	Describe the rationale for the review in the context of what is already known. Explain why the review questions/objectives lend themselves to a scoping review approach.	3-6
Objectives	4	Provide an explicit statement of the questions and objectives being addressed with reference to their key elements (e.g., population or participants, concepts, and context) or other relevant key elements used to conceptualize the review questions and/or objectives.	5-6
**METHODS**			
Protocol and registration	5	Indicate whether a review protocol exists; state if and where it can be accessed (e.g., a Web address); and if available, provide registration information, including the registration number.	7
Eligibility criteria	6	Specify characteristics of the sources of evidence used as eligibility criteria (e.g., years considered, language, and publication status), and provide a rationale.	7-8
Information sources*	7	Describe all information sources in the search (e.g., databases with dates of coverage and contact with authors to identify additional sources), as well as the date the most recent search was executed.	6
Search	8	Present the full electronic search strategy for at least 1 database, including any limits used, such that it could be repeated.	8
Selection of sources of evidence†	9	State the process for selecting sources of evidence (i.e., screening and eligibility) included in the scoping review.	7-8
Data charting process‡	10	Describe the methods of charting data from the included sources of evidence (e.g., calibrated forms or forms that have been tested by the team before their use, and whether data charting was done independently or in duplicate) and any processes for obtaining and confirming data from investigators.	7-8
Data items	11	List and define all variables for which data were sought and any assumptions and simplifications made.	7-8
Critical appraisal of individual sources of evidence§	12	If done, provide a rationale for conducting a critical appraisal of included sources of evidence; describe the methods used and how this information was used in any data synthesis (if appropriate).	N/A
Synthesis of results	13	Describe the methods of handling and summarizing the data that were charted.	8
**RESULTS**			
Selection of sources of evidence	14	Give numbers of sources of evidence screened, assessed for eligibility, and included in the review, with reasons for exclusions at each stage, ideally using a flow diagram.	9
Characteristics of sources of evidence	15	For each source of evidence, present characteristics for which data were charted and provide the citations.	11-21
Critical appraisal within sources of evidence	16	If done, present data on critical appraisal of included sources of evidence (see item 12).	N/A
Results of individual sources of evidence	17	For each included source of evidence, present the relevant data that were charted that relate to the review questions and objectives.	10-35
Synthesis of results	18	Summarize and/or present the charting results as they relate to the review questions and objectives.	10-35
**DISCUSSION**			
Summary of evidence	19	Summarize the main results (including an overview of concepts, themes, and types of evidence available), link to the review questions and objectives, and consider the relevance to key groups.	28-35
Limitations	20	Discuss the limitations of the scoping review process.	32-33
Conclusions	21	Provide a general interpretation of the results with respect to the review questions and objectives, as well as potential implications and/or next steps.	33-34
**FUNDING**			
Funding	22	Describe sources of funding for the included sources of evidence, as well as sources of funding for the scoping review. Describe the role of the funders of the scoping review.	38

JBI = Joanna Briggs Institute; PRISMA-ScR = Preferred Reporting Items for Systematic reviews and Meta-Analyses extension for Scoping Reviews.

* Where sources of evidence (see second footnote) are compiled from, such as bibliographic databases, social media platforms, and Web sites.

† A more inclusive/heterogeneous term used to account for the different types of evidence or data sources (e.g., quantitative and/or qualitative research, expert opinion, and policy documents) that may be eligible in a scoping review as opposed to only studies. This is not to be confused with information sources (see first footnote).

‡ The frameworks by Arksey and O’Malley (6) and Levac and colleagues (7) and the JBI guidance (4, 5) refer to the process of data extraction in a scoping review as data charting.

§ The process of systematically examining research evidence to assess its validity, results, and relevance before using it to inform a decision. This term is used for items 12 and 19 instead of"risk of bias"(which is more applicable to systematic reviews of interventions) to include and acknowledge the various sources of evidence that may be used in a scoping review (e.g., quantitative and/or qualitative research, expert opinion, and policy document).

From: Tricco AC, Lillie E, Zarin W, O'Brien KK, Colquhoun H, Levac D, et al. PRISMA Extension for Scoping Reviews (PRISMAScR): Checklist and Explanation. Ann Intern Med. 2018;169:467–473. https://doi.org/10.7326/M18-0850.

## Appendix B: Eligibility Flowchart

If unsure of any criterion, provisionally assume ‘yes’ to include for further review:

## Abbreviations

H&Y: Hoehn and Yahr
IPAQ: International Physical Activity Questionnaire
IPAQ-S: International Physical Activity Questionnaire Short-form
LSVT: Lee Silverman Voice Technique
METs: Metabolic equivalents
MIRT: Multidisciplinary Intensive Rehabilitation Treatment
MVPA: Moderate to vigorous physical activity
NICE: National Institute of Health and Care Research
PAPT: Proactive physical therapy
PCC Framework: Population, Concept, and Context Framework
PDDS: Parkinson’s Disease Disability Scale
PDQ-39: 39-Item Parkinson’s Disease Questionnaire
PRISMA-ScR: Preferred Reporting Items for Systematic Reviews and Meta-Analyses extension for Scoping Reviews
pwp: People with Parkinson’s disease
TIDieR: Template for Intervention Description and Replication
UPDRS: Unified Parkinson’s Disease Rating Scale
VO_2_ max: Maximal oxygen uptake
VT: Ventilatory threshold
WHO: World Health Organization
6MWT: 6 Minute Walk Test
