# Supplementary material for: Interventions for promoting physical activity in people with newly diagnosed Parkinson’s disease: scoping review
Source: Syst Rev. 2025 Aug 9;14:164. doi: 10.1186/s13643-025-02892-2 (PMC12335044; doi:10.1186/s13643-025-02892-2)
Supplement: Supplementary file 1 — Supplementary Material 1. Study characteristics. [file 13643_2025_2892_MOESM1_ESM.docx]

**Additional Information. Study characteristics**

| Author | Age (Yrs) | Gender (Male/ Female) | Time since diagnosis (Yrs) | Disease Severity and Medication Usage at Study Start | Other Participant Characteristics and Adherence | Type of Profession Delivering Intervention and Study Team Fidelity | Country/Setting(s) |
| --- | --- | --- | --- | --- | --- | --- | --- |
| Bang & Shin (2017). | Nordic walking training group: 58.3 (SD 7.71). Treadmill training group: 60.6 (SD 6.74). | Nordic walking training group: male: 5 (50%). Treadmill training group: male 4 (40%). | Nordic walking training group: 1.5 (SD 0.56). Treadmill training group: 1.5 (SD 0.27). | H & Y: 1 to 3.  Medication usage not reported. | No between-group differences in terms of sex, age, height, weight, MMSE, and duration of PD.  All participants completed all training and assessment sessions. | Physiotherapist.  Fidelity not reported. Evaluator experienced and qualified to use the measurement tools. | Republic of Korea.  Clinical setting. |
| Cancela-Carral *et al*. (2022). | 71.1 (SD 9.04). | Male 38 (53%). | 3.83 (SD 1.3) | H & Y 1 to 4  Medication usage not reported.  MDS-UPDRS mean 22.35 (SD 15.6). | 100 % Caucasian.  Education level (n, %) . No education: 12 (16.9%); Primary education: 40 (56.3%); Secondary education: 6 (8.4%); University education: 13 (18.3%).  BMI 2011: 26.62 (SD 3.36); 2015: 26.53 (SD 3.6); 2019: 26.46 (SD 3.64).  12 people failed to meet 80% of physical exercise program attendance by 2019. | Health professionals (physiotherapy graduates, physical activity, and sports science graduates).  Fidelity not reported. | Spain.  Community setting. |
| Carda *et al.* (2012). | Mean (SD) Experimental Group: 67.87 (7.05); Control Group 66.93 (5.13). | 17 (60.7%) male. | Mean (SD) Experimental Group 3.73 (2.49); Control Group 3.73 (1.91). | Mean (SD) Experimental Group H & Y 2.17 (0.24); Control Group H & Y 2.23 (0.26).  LDE (mg/day) Mean (SD) Experimental Group: 393.8 (165.7); Control Group: 371.4 (99.4). Patients excluded if they had current levodopa therapy that was started more than 6 months before enrolment. | MMSE Mean (SD) Experimental Group 25.94 (2.04); Control Group: 25.52 (2.19).  Patients had to have had no treadmill training or other form of specific gait training for at least 6 months before the study.  2 participants lost to follow-up | Sessions observed by physiotherapist.  Outcome measures recorded by a physiotherapist blinded to treatment allocation. | Switzerland.  Laboratory setting. |
| Clarke *et al*. (2016). | Intervention group: 70 (SD 9.1); Range 35-90. Control group: 70 (SD 9.3); Range 35-91. | Intervention group: male 240 (63%); Control group male 258 (68%). | Intervention group: 4.5 (SD 4.9). Control group: 4.6 (SD 4.5). | H & Y: 1 to 4.  LDE (mg/day): Intervention group n = 381; mean (SD) 453 (357.9); range 0-1877.  Control group n = 381; mean (SD) 498 (372.8) range 0-2181. | Other characteristics not reported.  In total, 25 patients (6%) allocated to PT and OT did not receive therapy by 3 months after randomization (12 started PT and/or OT after 3 months and 13 (3%) never received any therapy. Nine patients (2%) allocated to no therapy received therapy for PD-related problems within 3 months. | Physiotherapist and Occupational Therapist.  Lack of consistency in therapy assessment and intervention approaches used. | UK.  NHS outpatient clinics. |
| Ellis *et al*. (2019). | 64.1 (SD 9.5). | Male 28 (54.9%). | 4.8 (SD 3.1). | H & Y: 1 to 3.  Medication usage not reported. | Sample was highly educated, lacked racial diversity.  Exercise adherence data were collected via daily records of steps taken and exercises performed, using either the mobile health application (mHealth group) or paper calendars (active control group). Adherence similar between groups. Average number of days per week 3.2 mHealth cohort; 3.5 active control group. | Physiotherapist with expertise in Parkinson’s to deliver an individualised exercise and walking program (Exercise program from predetermined set based on APDA “Be Active and Beyond” program). | USA.  Free-living environment. |
| Fishel *et al.* (2020). | 69. | Male. | 2. | H & Y: 3.  On selegiline (dose not reported). | Participant 3: manager of a telecommunications company and lived in a single family home with his wife.  All sessions attended and all measures completed. Home Exercise Plan 80% adherence. | Occupational Therapist or Physiotherapist.  Fidelity not reported. | USA.  Outpatient Clinic within an academic setting. |
| Frazzitta *et al*. (2015). | 69 (SD 6). | Intervention group; male 45%. Control group: male 60%. | Intervention on diagnosis. | H & Y: 1 to 2.  All patients (n = 45 MIRT group; n = 60 control group) on rasagiline (dose not reported) and levodopa naïve. | Other characteristics not reported.  16 completed. On average, patients in MIRT Group carried out 95% of the scheduled MIRT sessions. | Neurologists, Physiatrists, Psychologists, Nurses, Physiotherapists, and Occupational Therapists.  Fidelity not reported. | Italy.  Ward setting. |
| Handlery *et al*. (2021). | Median 65 (range 40-80). | Male 62 (56.4%); female 48 (43.6%). | Total: Median 0 (range 0-5). Participants with >4200 steps/day (n=74) Median 0 (range 0-5). Participants with <4200 steps/day (n=36) Median 1 (range 1-5). | H & Y: < 3.  Dopaminergic therapy naiive.  MDS-UPDRS motor score (part III) median 19 (range 5-60); | Fear/worry of falling. Never: n = 77 (70%). Any: n = 33 (30%).  Cardiovascular condition present: n = 52 (47.3%).  Baseline VO₂ max (ml/min/kg): median 22.8 (range 9.2-43.7).  Met aerobic PA guidelines at baseline: n = 84 (76.4%).  Targeted treadmill intensity; targeted treadmill session number; steps per session; mean activity monitor wear time (809mins (SD 87)). | Study Coordinators from: School of Physical Therapy, Arkansas College of Health Education; Department of Exercise Science and the Department of Health Promotion, Education, and Behaviour University of South Carolina.  Protocol fidelity was ensured by monthly conference calls with study coordinators. | USA.  Outpatient clinics and community-based exercise facilities. |
| van der Kolk *et al*. (2019). | Intervention group: 59.3 (SD 8.3). Control group: 59.4 (SD 9.3). | Intervention group: male 42 (65%)/female 23 (35%); Control group: male 38 (58%)/ female 27 (42%). | Intervention group median: 3.4 (IQR 1.3 - 7.3). Control group median:3.2 (IQR 1.6 - 6.8). | H & Y: 1 to 2.  Patients on dopaminergic therapy: Intervention n= 61 (94%); control n = 63 (97%).  LDE (mg): Intervention 600 (range 375-890).  Control 532 (range 300- 838). | Educated > 15 yrs; 84% were married; 30% still working.  20 patients (10 in each group) did not complete their allocated exercise programme but attended the post-intervention visit after 6 months and were therefore included in the data analysis. Total attrition rate of 19%: 22% for aerobic exercise and 17% for active controls. | Coach (Physical therapists or Research Assistant).  Fidelity not reported. | Netherlands.  Free-living environment. |
| Landers *et al*. (2019). | Intervention mean 63.5 (SD 10.9); Control mean 64.6 (SD 6). | Intervention: male n = 10 (71%); Control: male n = 9 (69%). | Intervention mean 4.92 (SD 5.1); Control mean 4.7 (SD 3.9). | Intervention H&Y1: n = 2; H&Y2: n = 10; H&Y3: n = 2. Control H&Y1: n = 3; H&Y2: n = 7; H&Y3: n = 3.  LDE (mg/day): Intervention mean 419.3 (SD 389.2);  Control mean 476.7. (SD 300).  MDS-UPDRS III at baseline (on-medication) mean (SD): Intervention: 27.5 (10.7) Control: 36.5 (15). | Excluded regular exercisers (≥3 exercise bouts per week that would produce >60% of estimated maximum heart rate).  Attendance (at least 3 sessions per week): Intervention: 11 of 13; Control: 7 of 11.  Aerobic intensity (150mins moderate intensity exercise at 70% estimated maximum heart rate) Intervention: 8 of 13; Control: 2 of 11.  Strength training target (2 days per week) Intervention: all; Control : none.  Attrition: 3 of 27 (Intervention n = 1; Control n = 2). | Physiotherapists.  Protocol fidelity was addressed through: pretrial training sessions, standardized protocol manual, protocol flowsheets in participant charts, and e-mail/phone reminders. | USA.  Gym environment. |
| Leavy *et al*. (2019). | 70 to 84. | 3 male/ 5 female (sub-category of <5 yrs since diagnosis). | 1 to 5. | H & Y: 2 to 3.  Medication usage not reported. | MMSE: 25-29.  All completed semi-structured interviews. | Physiotherapists. All educated regarding the program (Hi-Balance programme); Qualitative researcher with semi-structured interviews (this article). | Sweden.  Free-living environment. |
| Li *et al.* (2020). | 71.1 (SD 8.2) | Male: 108 (71%). | 3.1 (SD 0.6 - 6.6) | H&Y 1: 80 (54%); H&Y 2: 44 (30%); H&Y 3: 23 (15%); H&Y 4: 2 (1%).  LDE mg/day): 600 (300 - 825). | Retired 83%.  Preferred language English 94 %.  Comorbidities: 0: 7%; 1: 18%; 2+: 74%.  Exerciser: 16%.  MMSE: median 27 (27-30).  Fell in past yr: 47%.  FOG: 43%.  BBS: median 56 (52-56).  PD Fatigue Scale: mean 46 (SD 16.3).  DASS-21: no stress 80%.  16 participants lost to follow-up. | Multidisciplinary team including a physiotherapist, exercise physiologist, rehabilitation specialist, dietician, speech pathologist and social worker (Exercise component delivered by a physiotherapist and exercise physiologist).  Fidelity not reported. | Australia.  Hospital outpatient setting. |
| Moriello *et al*. (2013). | 57. | Male. | 2. | Disease severity not reported.  Stalevo 150mg 6 times/day, azilect 1mg daily, Myoplex 0.5mg 3 times/day. | Working full time. Hobbies of running, biking, water skiing, snow skiing, dancing severely restricted.  Phase A (0-12 weeks) supervised; Phase B (Home exercise Plan - participant reported). | Physiotherapist.  Fidelity not reported. | USA.  Outpatient clinic and free-living environment. |
| Nero *et al*. (2016). | Training group: 72 (SD 6). Control group: 74 (SD 6). | Training Group: male 27 (63%)/ female 16 (37%); Control Group: male 20 (50%)/female 20 (50%). | Training group median (IQR): 5 (2-10). Control group: 5 (2-8). | H & Y: 2 to 3.  LDE (mg/day): Intervention group n = 43; mean (SD) 584 (290); control group n = 40; mean (SD) 649 (420). | Other characteristics not reported.  Of the 100 included and randomized participants, 76 completed the final 12 month follow-up. Only 66 participants contributed with PA-data at 12 months, due to invalid or missing accelerometer data. | Physiotherapists.  All educated regarding the Hi-Balance program. | Sweden.  Free-living environment. |
| Penko *et al*. (2021). | Forced Exercise 63 (SD 8); Voluntary Exercise 61 (SD 9); Control: 65 (SD 6). | Forced Exercise male 19 (54%); Voluntary Exercise male 19 (54%); Control male 14 (70%). | Forced Exercise Median 3 (IQR 1-3); Voluntary Exercise Median 2 (IQR 2-5); Control Median 3 (IQR 1-3). | UPDRS III Forced Exercise Mean 36.11 (SD 8.95); Voluntary Exercise Mean 39.47 (SD 9.54); Control Mean 34.5 (SD 12.27).  LDE mg/day Forced Exercise Median 450 (IQR 210-600); Voluntary Exercise Median 450 (IQR 300-665); Control Median 450 (IQR 145-562). | Other characteristics not reported.  Exercise attendance, (defined as completion of the 24 exercise sessions within 9 weeks) Forced Exercise group: 97%; Voluntary Exercise Group : 93%. | Exercise physiologist.  American Heart Association/American College of Sports Medicine test termination criteria used. | USA.  Research laboratory. |
| Rafferty *et al*. (2019). | 65.1 (SD 11.4) (Range 43 to 83). | Male 16 (57%). | 0.1 to 5. | H & Y: 1 to 2.  Medication (dosage not reported): none n = 12 (43%); carbidopa/ levodopa only n = 10 (36%); MAOB-inhibitor only n = 4 (14%); 2+ medications n = 2 (7%). | Work status: Full-time employment n = 12 (43%); Retired n = 13 (46%); Disability n = 1 (4%); Unknown n = 2 (7%). All with private health insurance.  Participant adherence not reported. | Physiotherapists  Co-treated the first referred patient and then met intermittently through the year to review assessments, resources, and standardised delivery components. | USA  Outpatient clinic and free-living environment. |
| Schenkman *et al*. (2012). | AE: 63.4 (SD 11.2).  FBF: 64.5 (SD 10). Control group: 66.3 (SD 10.1). | Control group: male 26 (63.4%); AE male 26 (63.4%); FBF male 24 (61.5%). | Control mean 4.5 (SD 3.8); AE mean 3.9 (SD 4.2); FBF mean 4.9 (SD 3.7). | H & Y: 1 to 3.  Medication usage not reported. | Majority of participants were men, married, retired, and with an annual income of more than USD$50,000.  86.8%, 82.6%, and 79.3% completed 4, 10, and 16months, respectively, of the intervention. No reliable data on participants’ home exercise adherence - Exercise diary accuracy was insufficient for meaningful interpretation. | Physiotherapists.  All personnel who supervised the exercise sessions were trained by the primary investigator, received written materials outlining the exercise protocols in detail and co-treated with the primary investigator periodically to ensure consistency. | USA.  Outpatient clinics within an academic setting/ Community facility and free-living environment. |
| Schenkman *et al*. (2018) | 64 (SD 9, range, 40-80). | Male 73 (57.0%). | High-Intensity Exercise group: median ( IQR) 0.3 (0.1-1.3). Moderate-Intensity Exercise group: median (IQR) 0.3 (0.2-0.8); Usual Care group: median (IQR) 0.4 (0.1- 0.8). | H & Y: 1 to 2.  All participants levodopa naïve. | Majority of participants were non-Hispanic white (n = 108 (84.4%)).  MOCA score mean: 28  Adherence was determined by exercise frequency. Participants with Parkinson disease adhered to the prescribed exercise intensity and met the hypothesized frequency of 3 days per week during 6 months. | Study Coordinators.  Protocol fidelity was ensured by monthly conference calls with study coordinators. | USA.  Outpatient clinics and community-based exercise facilities. |
| So *et al*. (2023). | Intervention: 59.07 (SD 6.10); Control: 59.35 (SD 5.92). | Intervention: male: 8 (53.3%); Control male 8 (47.1%). | Intervention: 1.40 (SD 0.89); Control: 0.98 (SD 1.11). | Intervention: H & Y 1: n = 2 (13.3%); H & Y 2: n = 13 (86.7%). Control: H & Y 1: n = 2 (11.8%); H & Y 2: n = 15 (88.2%).  LDE mg/day: Intervention: mean 374.17 (SD 142.56); Control: mean 294.18 (SD 163.00).  MDS-UPDRS III: Intervention: mean 22.07 (SD 6.86); Control mean 21.06 (SD 7.73). | Educated (80% high-school/college educated.  > 70% participants family income > $2600 dollars per month.  Participants were active at baseline (> 2000 MET minutes per week).  36% dropout rate; Attendance rate for telephone counselling and coaching was 80-100%. | Community Health Nurses.  Outcomes collected by coordinator nurse - no further details. | Republic of Korea.  Community setting. |
| Solla *et al*. (2019). | Intervention group mean 67.8 (SD 5.9); Control group mean 67.1 (SD 6.3). | Intervention group: male 6 (60%); Control group: male 7 (70%). | Intervention group mean 4.4 (SD 4.5); Control group mean 5 (SD 2.9). | H&Y Intervention group mean 2.1 (SD 0.6); Control group mean 2.3 (SD 0.4).  LDE mg/day Intervention group mean 481.1 (SD 213.1); Control group mean 487.5 (SD 198.5). | Participant attendance at the dance classes during the program 92.9%. | Adapted Physical Activity Postgraduate students and a sports association that promotes exercise therapy.  Study team fidelity not reported. | Italy.  Outpatient health clinic. |
| Tucak *et al*. (2023). | Age at entry mean: 64.3 (SD 9.6). Age range 47 – 80. | Male 16 (94.1%). | Mean: 4.3 (SD 2.9). | H&Y 1: n = 6 (35.3%); H&Y 2: n = 10 (58.8%); H&Y 3: n = 1 (5.9%).  LDE mg/day: 527.2 (SD 413.2).  MDS-UPDRS mean 22.5 (SD 14.4). | Other characteristics not reported.  3 lost to follow-up. Overall attendance rate of 93% at the class sessions. | PDW trained exercise instructor. | Australia.  Outpatient health clinic. |
| Vistven *et al*. (2023). | 49 to 74. | 5 female, 2 male. | 2 to 4 years. | H & Y 1.5 to 2.  Medication usage not reported. | 2 out of 7 participants in employment.  5 out of 7 participants inpatients. | Tailored MDT including physiotherapists, team nurses and neurologists.  Assessments conducted by physiotherapists.  First author a physiotherapist - completed inductive analysis. Second author proposed analytical questions. Preliminary results presented in research seminars to colleagues and main themes reduced from five to three. | Norway.  Intervention provided in inpatient and outpatient settings.  Interviews conducted in participant homes |
| Key: data presented as mean and standard deviation (SD) unless stated otherwise; AE = Aerobic Exercise; APDA = American Parkinson’s Disease Association; BMI = Body Mass Index; DASS-21 = Depression, Anxiety and Stress Scale; FBF = Flexibility/Balance/Functional training; FOG = Freezing of Gait; IQR = Interquartile range; H & Y = Hoehn and Yahr Stage; LDE = levodopa dose equivalent; MAOB = monoamine-oxidase B inhibitors; MDS-UPDRS = Movement Disorder Society Unified Parkinson’s Disease Rating Scale; mg = milligrams; MET = Metabolic Equivalent of Task; MIRT = multidisciplinary intensive rehabilitation treatment; MMSE = mini-mental state examination; n = number; NHS = National Health Service; OT = Occupational Therapy; PA = Physical Activity; PD = Parkinson’s Disease; Yr(s) = year(s); VO₂ max (ml/min/kg) = Maximum Volume of Oxygen in milliliters per minute per kilogram. | | | | | | | |
